# A novel AMPK activator shows therapeutic potential in hepatocellular carcinoma by suppressing HIF1α‐mediated aerobic glycolysis

**DOI:** 10.1002/1878-0261.13211

**Published:** 2022-04-12

**Authors:** Hsing‐I Tseng, Yi‐Siang Zeng, Ying‐Chung Jimmy Lin, Jui‐Wen Huang, Chih‐Lung Lin, Meng‐Hsuan Lee, Fan‐Wei Yang, Te‐Ping Fang, Ai‐Chung Mar, Jung‐Chen Su

**Affiliations:** ^1^ Department of Pharmacy National Yang Ming Chiao Tung University Taipei Taiwan; ^2^ Institute of Biopharmaceutical Sciences National Yang Ming Chiao Tung University Taipei Taiwan; ^3^ Department & Institute of Physiology National Yang Ming Chiao Tung University Taipei Taiwan; ^4^ 33561 Department of Life Science and Institute of Plant Biology National Taiwan University Taipei Taiwan; ^5^ 33561 Genome and Systems Biology Degree Program National Taiwan University and Academia Sinica Taipei Taiwan; ^6^ 63129 Biomedical Technology and Device Research Labs Industrial Technology Research Institute Hsinchu Taiwan; ^7^ Taiwan International Graduate Program in Molecular Medicine National Yang Ming Chiao Tung University and Academia Sinica Taipei Taiwan

**Keywords:** AMPK, hepatocellular carcinoma, HIF1α, SCT‐1015

## Abstract

Hepatocellular carcinoma (HCC) is characterized by rapid growth, early vascular invasion, and high metastasis. Currently available US Food and Drug Administration (FDA)‐approved drugs show low therapeutic efficacy, limiting HCC treatment to chemotherapy. We designed and synthesized a novel small molecule, SCT‐1015, that allosterically activated adenosine monophosphate‐activated protein kinase (AMPK) to suppress the aerobic glycolysis in HCC. SCT‐1015 was shown to bind the AMPK α and β‐subunit interface, thereby exposing the kinase α domain to the upstream kinases, resulting in the increased AMPK activity. SCT‐1015 dramatically reduced HCC cell growth *in vitro* and tumor growth *in vivo*. We further found that AMPK formed protein complexes with hypoxia‐inducible factor 1‐alpha (HIF1α) and that SCT‐1015‐activated AMPK promoted hydroxylation of HIF1α (402P and 564P), resulting in HIF1α degradation by the ubiquitin‐proteasome system. With declined HIF1α abundance, many glycolysis‐related enzymes were downregulated, suppressing aerobic glycolysis, and promoting oxidative phosphorylation. These results indicated that SCT‐1015 channeled HCC cells into an unfavorable metabolic status. Overall, we reported SCT‐1015 as a direct activator of AMPK signaling that held therapeutic potential in HCC.

## Graphical abstract

In HCC, the transcription factor HIF1α regulates the expression of HK and PDK, leading to the Warburg phenotype, which promotes HCC progression. Under treatment with SCT‐1015, a novel AMPK activator, the HIF1α protein abundance was reduced through the increased HIF1α protein degradation (ERK/PHD‐dependent HIF1α hydroxylation) and the decline of the HIF1α protein synthesis (mTORC1 signaling), eliciting an anti‐Warburg effect and an anti‐HCC effect.

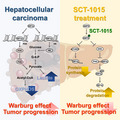

**Abbreviations**
2‐DG: 2‐deoxyglucose
AMPK: adenosine monophosphate‐activated protein kinase
CaMKKβ: Ca^2+^/CaM‐dependent protein kinase Cam β
CBM: carbohydrate‐binding module
CE‐MS: capillary electrophoresis mass spectrometry
CHX: cyclohexamide
DME: dimethyoxyethane
ECAR: extracellular acidification rate
ERK: extracellular regulated kinase
F1,6BP: fructose 1,6‐bisphosphate
F6P: fructose 6‐phosphate
G6P: glucose 6‐phosphate
HCC: hepatocellular carcinoma
HEXO 2: hexokinase 2
HIF1α: hypoxia‐inducible factor 1‐alpha
IHC: immunohistochemistry
LDHA: lactate dehydrogenase A
LKB1: liver kinase B1
mTOR: mammalian target of rapamycin
OS: overall survival
OXPHOS: oxidative phosphorylation
pβAMPK: phosphorylated AMPK
PDH: pyruvate dehydrogenase
PFKP: plateletβtype phosphofructokinase
PGAM1: phosphoglycerate mutase
PHD: prolyl hydroxylases
PLC5: PLC/PRF/5
qPCR: quantitative PCR
shRNAs: short hairpin RNAs
TAK1: transforming growth factor betaβactivated kinase 1

## 1 Introduction

Hepatocellular carcinoma (HCC) ranks as the sixth most common solid tumor worldwide and the fourth leading cause of cancer‐related death [1]. HCC shows rapid growth and an early vascular invasion property with poor prognosis, a high tendency for metastasis, high mortality, and incidence rates [2, 3]. Currently, four US Food and Drug Administration (FDA)‐approved small molecules (sorafenib, regorafenib, lenvatinib, and cabozantinib) for HCC treatment are multiple receptor tyrosine kinase inhibitors and showed limited therapeutic efficacy in the treatment of HCC patients. For example, sorafenib, the first‐line drug for HCC treatment, only prolongs median overall survival (OS) up to 2 to 3 months in HCC patients [4]. Therefore, it is important to consider new therapeutic approaches, including new biomarkers targeting drug development for survival improvement in HCC patients.

The metabolic flux preference for energetic production in cancer is significantly different from normal cells. Normal cells process glucose through glycolysis, transfer pyruvate into mitochondria, and subsequently generate ATP through oxidative phosphorylation (OXPHOS) in aerobic conditions. Under anaerobic conditions, normal cells increase the lactate‐to‐pyruvate ratio and generate much less ATP than OXPHOS. Compared with normal cells, cancer cells uptake more glucose and increase lactate production regardless of oxygen concentration, which is commonly accompanied by OXPHOS reduction. Such metabolic reprogramming (the increased aerobic glycolysis and the reduced OXPHOS status) is defined as the ‘Warburg effect’ [5] and has been found to promote tumorigenesis [6]. Several oncogenes or tumor suppressors were observed to control this metabolic switch in cancer. For example, adenosine monophosphate‐activated protein kinase (AMPK), functions as a tumor suppressor, and negatively regulates the Warburg phenotype in myc‐driven lymphomagenesis [7].

AMPK, a heterotrimeric complex, is composed of catalytic α, regulatory β, and γ subunits [8]. Each subunit contains two to three isoforms (α1, α2, β1, β2, γ1, γ2, γ3) [9] and is expressed in many tissues, including liver [10]. AMPK has been known to play a crucial role in maintaining the metabolic energy homeostasis and is regulated by the cellular AMP/ATP ratio. Under an increased AMP/ATP ratio, AMP binds to the cystathionine β‐synthase domains of the AMPKγ subunit, and then the autoinhibitory domain of the α subunit rotates back behind the alpha‐kinase domain to further expose the catalytic site of the α subunit [11, 12]. This catalytic site would then be phosphorylated on the conserved threonine residue (Thr172) by several upstream protein kinases (AMPKK), such as tumor suppressor liver kinase B1 (LKB1) in complex with STRAD and MO25 [13, 14], Ca^2+^/CaM‐dependent protein kinase β (CaMKKβ) [13], or transforming growth factor beta‐activated kinase 1 (TAK1) [14] to activate AMPK. In HCC, the phosphorylated AMPK (p‐AMPK) abundance is relatively low in tumor sections compared with the adjacent nontumor HCC tissue sections [15]. Low AMPK phosphorylation status is correlated with poor OS and time to recurrence in HCC patients [15]. However, the role of AMPK in HCC metabolic reprogramming still needs to be elucidated.

Recently, numerous AMPK activators exhibited anticancer potential. Metformin, an approved type‐2 diabetes drug, has been found to attenuate tumor progression in xenograft mouse model and naturally arising lymphomas [16, 17]. Phenformin and buformin, structurally similar to metformin, also showed the potential for cancer treatment in numerous cancer types [18, 19]. Both A‐769662 and 991 are direct AMPK activators through allosteric interaction with carbohydrate‐binding module (CBM) of the β subunit and the kinase domain of the α subunit [20]. Nilotinib, the bcr‐abl kinase inhibitor, was approved for the treatment of imatinib‐resistant chronic myelogenous leukemia, and can induce autophagic cell death in HCC through AMPK activation [21]. These studies provided evidence for the potential use of AMPK activators in cancer therapy.

In this study, we developed a novel AMPK activator, SCT‐1015, which is structurally similar to nilotinib. We found that SCT‐1015 reduced aerobic glycolysis and enhanced the OXPHOS flux through downregulating the AMPK‐dependent hypoxia‐inducible factor 1‐alpha (HIF1α)‐mediated signaling pathway in HCC. Such redirection of metabolic status retarded the HCC cell growth and colony formation *in vitro*. For *in vivo* antitumor properties, SCT‐1015 elicited a more potent tumor‐suppressive effect than metformin in the PLC5 subcutaneous model. In addition, AMPK‐reduced HIF1α protein abundance through proline hydroxylation triggered ubiquitin‐proteasome degradation, which was reflected to the inverse correlation between AMPK and HIF1α in HCC cells and patient specimens. Our results demonstrated novel insights into the role of AMPK and HIF1α in HCC metabolic reprogramming and provided the therapeutic possibility through AMPK activation by our newly developed small molecule, SCT‐1015.

## 2 Materials and methods

### 2.1 Synthesis, purification, and characterization of SCT‐1015

***tert*‐butyl 2‐(2‐chloropyrimidin‐4‐yl)‐1*H*‐pyrrole‐1‐carboxylate (3)**. Trimethylamine (1.2 mL, 8.6 mmol, 5.4 equiv.) was added to the solution containing 2,4‐dichloropyrimidine **(1)** (236 mg, 1.6 mmol, 1.0 equiv.), *N*‐Boc‐2‐pyrroleboronic acid **(2)** (400 mg, 1.9 mmol, 1.2 equiv.), Pd(dppf)Cl_2_・CH_2_Cl_2_ (100 mg, 0.1 mmol, 0.08 equiv.), and dimethyoxyethane (DME) (2 mL). The mixture was stirred at 90 °C for 16 h, and then a celite pad was used for filtration. The filtrate was extracted several times using ethyl acetate/H_2_O, and the organic part was concentrated. Purification by column chromatography (hexane/ethyl acetate 17 : 3) was used to obtain **(3)** in 46% yield. ^1^H NMR (400 MHz, CDCl_3_): δ 8.47 (d, *J* = 5.2 Hz, 1H), 7.36 (dd, *J* = 3.2, 2.0 Hz, 1H), 7.27 (d, *J* = 5.2 Hz, 1H), 6.64 (dd, *J* = 3.6, 1.6 Hz, 1H), 6.20 (t, *J* = 3.6 Hz, 1H), and 1.40 (s, 9H) p.p.m. **1‐(4‐chloro‐3‐(trifluoromethyl)phenyl)‐3‐(3‐nitrophenyl)urea (6)**. The triphosgene (645 mg, 2.2 mmol, 1.0 equiv.) was added to dry tetrahydrofuran (2 mL), and solution A was cooled to 0 °C. Then 3‐nitroaniline **(5)** (300 mg, 2.2 mmol, 1.0 equiv.), and trimethylamine (0.64 mL, 4.6 mmol, 2.1 equiv.) were added into dry tetrahydrofuran (10 mL) to form solution B. Solution B was slowly added to solution A, and then the mixture was stirred at 40 °C for 30 min. 4‐chloro‐3‐(trifluoromethyl)aniline **(4)** (425 mg, 2.2 mmol, 1.0 equiv.) was slowly added to the mixture and stirred at 45 °C for 16 h. This mixture was filtered, and the precipitate was rinsed with water and acetone to obtain **(6)** in 19% yield. ^1^H NMR (400 MHz, MeOD‐*d_4_*): δ 8.51 (s, 1H), 8.01 (s, 1H), 7.90 (d, *J* = 7.6 Hz, 1H), 7.78 (d, *J* = 7.6 Hz, 1H), 7.68 (d, *J* = 8.0 Hz, 1H), and 7.56 – 7.52 (m, 2H) p.p.m. **1‐(3‐aminophenyl)‐3‐(4‐chloro‐3‐(trifluoromethyl)phenyl)urea (7)**. Compound **(6)** (500 mg, 1.4 mmol, 1 equiv.) in ethyl acetate with 10% Pd/C and H_2_ gas was stirred for 16 h. The mixture was filtered through a celite pad, and the filtrate was concentrated. Column chromatography (hexane/ethyl acetate 13 : 9) was applied to purify and to obtain **(7)** in 55% yield. ^1^H NMR (400 MHz, MeOD‐*d_4_*): δ 7.98 (d, *J* = 2.4 Hz, 1H), 7.60 (dd, *J* = 8.8, 2.4 Hz, 1H), 7.49 (d, *J* = 8.8 Hz, 1H), 7.02 (t, *J* = 8.0 Hz, 1H), 6.91 (t, *J* = 2.0 Hz, 1H), 6.70 (dd, *J* = 8.0, 1.2 Hz, 1H), and 6.44 (dd, *J* = 8.0, 1.2 Hz, 1H) p.p.m. **1‐(3‐((4‐(1*H*‐pyrrol‐2‐yl)pyrimidin‐2‐yl)amino)phenyl)‐3‐(4‐chloro‐3‐(trifluoromethyl)phenyl)urea (SCT‐1015)**. Three drops of 37% HCl were added to the isopropyl alcohol (4 mL) solution with compound **(7)** (70 mg, 0.21 mmol, 1 equiv.) and compound **(3)** (71 mg, 0.25 mmol, 1.2 equiv.). The mixture was stirred at 100 °C for 16 h and then filtered, and the precipitate was rinsed with ethyl acetate to obtain **SCT‐1015** in 37% yield. ^1^H NMR (400 MHz, DMSO‐*d_6_*): δ 11.75 (s, 1H), 10.55 (s, 1H), 9.91 (s, 1H), 9.61 (s, 1H), 8.45 (s, 1H), 8.35 (d, *J* = 6.4 Hz, 1H), 8.08 (s, 1H), 7.64 (s, 2H), 7.38 (s, 1H), 7.34 (d, *J* = 6.4 Hz, 1H), 7.28 (t, *J* = 8.0 Hz, 1H), 7.22 (s, 1H), 7.12 (d, *J* = 8.0 Hz, 1H), 7.03 (d, *J* = 8.0 Hz, 1H), and 6.37 – 6.35 (m, 1H) p.p.m. ^13^C NMR (100 MHz, DMSO‐*d_6_*): δ 159.5, 153.1, 152.3, 148.3, 139.2, 138.8, 137.8, 131.6, 128.8, 127.9, 126.8, 126.3 (q, *J* = 30.4 Hz), 122.5, 122.3 (q, *J* = 271.3 Hz), 121.9, 116.7, 116.1 (q, *J* = 5.5 Hz), 113.7, 113.1, 111.4, and 109.8, 104.7 p.p.m. HRMS was calculated for C_22_H_16_ClF_3_N_6_O (M‐H)^‐^: 471.0942. Found: 471.0957. The synthetic steps and spectra are shown in Fig. S1 and S2.

### 2.2 Molecular docking

The protein X‐ray structure of AMPK (PDB id: 4CFE) with a resolution of 3.02 Å was a receptor retrieved from the Protein Data Bank [22]. The binding site was defined according to the coordinates of its crystal ligand, a benzimidazole derivative (991). All chemical structures for the docking were drawn in MarvinSketch and then were prepared using the ligand preparation module of Sybyl‐X. Molecular docking studies were carried out using GOLD (Genetic Optimization for Ligand Docking) 5.2.2 from the Cambridge Crystallographic Data Center [23]. The docking procedures, such as protein selection and setup, binding site definition, picking ligands, and choosing score function, were carried out by GOLD wizard. The binding site with a 10 Å radius sphere was defined around the ligand within the crystal structure. The default genetic algorithm settings were applied for all calculations, and the top 5 docked poses were saved for each ligand. The CHEMPLP score function was used to obtain the GOLD fitness score, which was calculated from the contributions of bonding interactions between the protein and ligands.

### 2.3 Cell culture

The Huh7 HCC cell line was obtained from the Health Science Research Resources Bank (HSRRB, Osaka, Japan). The PLC/PRF/5 (PLC5), Hep G2, SK‐HEP‐1, and Hep 3B cell lines were obtained from the American Type Culture Collection (ATCC, Manassas, VA, USA). The HA22T/VGH and HA59T/VGH cell lines were obtained from the Bioresource Collection and Research Center (BCRC, Hsinchu, Taiwan). All cells obtained from HSRRB, ATCC, or BCRC were immediately expanded and frozen such that all cell lines could be restarted every 3 months from a frozen vial of the same batch of cells.

### 2.4 Antibodies

Antibodies for immunoblotting such as p‐AMPKα (#2535), AMPKα (#5831), PDK1 (#3820), HIF1α (#3716), hexokinase 2 (#2867), LDHA (#3582), PFKP (#8164), PGAM1 (#12098), PDH (#3205), p‐Threonine/Tyrosine (#9381), p‐S6K (#9234), S6K (#2708), p‐ERK (#4370), ERK (#4695), p‐4E‐BP1 (Thr37/46, #2855), p‐4E‐BP1 (Ser65, #9451), 4E‐BP1 (#9644), mouse antirabbit IgG mAb (#5127), and antirabbit IgG (#7074) were from Cell Signaling Technology (Danvers, MA, USA). HIF1α (hydroxyl P564) (ab72777) and PHD3 (ab30782) were purchased from Abcam (Cambridge, MA, USA). HIF1α (hydroxyl P402) (07‐1585) was purchased from Merck (Darmstadt, Germany). GAPDH (sc‐25778) was purchased from Santa Cruz Biotechnology (Paso Robles, CA, USA). Actin (GTX109639) was purchased from GeneTex International (Hsinchu, Taiwan). Antibodies for immunoprecipitation such as HIF1α (#36169), rabbit mAb IgG (#3900), and p‐AMPKα (#2535) were purchased from Cell Signaling Technology. AMPKα1 (sc‐398861) was purchased from Santa Cruz Biotechnology. Antibodies for IHC such as p‐AMPKα (GTX52341) and HIF1α (GTX127309) were purchased from GeneTex International.

### 2.5 Plasmids

Short hairpin RNAs (shRNAs), including the control (shNT) and AMPKα1 (shAMPKα1), were purchased from BIOTOOLS (New Taipei, Taiwan). The constitutively active AMPKα1 construct, pEBG‐AMPKα1^1‐312^ (csAMPK), was purchased from Addgene (Addgene plasmid # 27632, Watertown, MA, USA) [24]. AMPKα2 expression plasmid was purchased from Origene (RC210226, Rockville, MD, USA). The HIF1α expression plasmid was purchased from Sino Biological (HG11977‐CM, Wayne, PA, USA). AMPKα1 and PDK1 were constructed into the pFLAG‐Myc‐CMV‐22 vector.

### 2.6 Abl1 kinase assay

Dose–response studies were performed with 10 concentrations in a 2‐fold dilution series from a maximum final compound concentration of 10 μm in the reaction mixture and were conducted at Reaction Biology (Malvern, PA, USA).

### 2.7 PP2A Phosphatase activity assay

The PP2A phosphatase activity was measured with the PP2A Immunoprecipitation Phosphatase Assay Kit (17‐313, Merck). PLC5 cells were seeded at a density of 8 × 10^5^ cells in 100‐mm dishes. After 24‐h incubation, cells were treated with the indicated dosage of compounds or DMSO in 5% FBS supplemented medium for 24 h. Cell extracts were prepared in Pierce IP Lysis buffer (87788, Thermo Fisher Scientific, Waltham, MA, USA) supplemented with protease inhibitors (10 μg·mL^−1^ aprotinin (A1153, Sigma‐Aldrich, St. Louis, MO, USA), 10 μg·mL^−1^ Leupeptin (L2884, Sigma‐Aldrich), 10 μg·mL^−1^ antipain (A6191, Sigma‐Aldrich), 10 μg·mL^−1^ soybean trypsin inhibitor (T9003, Sigma‐Aldrich), 10 μg·mL^−1^ Pepstatin A (P5318, Sigma‐Aldrich), 1 mm benzamidine (B6506, Sigma‐Aldrich), and 1 mm PMSF (14333, Cayman Chemical, Ann Arbor, MI, USA). The 200 μg cell extracts were incubated with 4 μg anti‐PP2Ac subunit antibody and 25 μL protein A agarose slurry for 2 h at 4 °C with gentle rotation. The PP2Ac‐containing complex beads were washed with 700 µL TBS three times, and then one wash with 500 µL pNPP Ser/Thr assay buffer. The PP2Ac‐containing complex beads were then incubated with 750 µm threonine phosphopeptide (K‐R‐Pt‐I‐R‐R) for 10 min at 30 °C with gentle rotation. After centrifugation, the 25 μL supernatant was transferred to the 1/2 volume microtiter plate. For the phosphate standard curve establishment, 25 µL of each phosphate standard (0, 200, 400, 600, 800, 1000, 1200, 1400, 1600, 1800, and 2000 picomoles of phosphate per 25 µL) was transferred to the 1/2 volume microtiter plate. Distilled water was used as a blank. The 100 μL malachite green detection solution was then added and incubated for 15 min at room temperature. The free phosphates were measured by optical density at 650 nm. All the PP2A phosphatase activity signals were first normalized by the blank signal. The amount of released phosphate could then be quantified by the phosphate standard curve.

### 2.8 Measurement of cell viability

The 2.5 × 10^3^ cells·well^−1^ in 5% FBS supplemented medium was seeded into 96‐well plates. After 24‐h incubation, the indicated dosages of compounds or DMSO were treated for 48 or 72 h. The cell viability was then assayed by PrestoBlue Cell Viability Reagent (A13262, Thermo Fisher Scientific) according to the manufacturer’s manual.

### 2.9 Colony formation assay

Cells were seeded at a density of 1.5 × 10^6^ cells in 100‐mm dishes. After 24 h incubation, cells were treated with the indicated dosages of compounds or DMSO in a 5% FBS supplemented medium for 48 h. Then 3 × 10^3^ treated cells were reseeded into 60‐mm dishes, and the 10% FBS supplemented medium was replaced every 3 days over 2 weeks. On day 14, the plates were washed in 1× PBS (phosphate‐buffered saline), fixed with 100% methanol and stained with a filtered solution of crystal violet (5% wt/vol). After washing with tap water, the colonies were counted using MetaMorph (Molecular Devices, San Jose, CA, USA).

### 2.10 Cytotoxic cellular assay

The 2.5 × 10^3^ cells·well^−1^ in 5% FBS supplemented medium was seeded into 96‐well plates. After 24 h incubation, the indicated dosages of SCT‐1015 or DMSO were treated for 48 h. Triton‐X 0.1% was used as a positive control. After the treatment, 20 µL of cell supernatant was transferred to white‐walled luminescence‐compatible 96‐well plates. The AK detection reagent reaction buffer (100 μL) from a ToxiLight bioassay kit (LT07‐217, Lonza, Basel, Switzerland) was added to each well. Luminescence was measured with a Spark 10 m multimode microplate reader (Tecan, Männedorf, Switzerland) after incubation for 5 min in the dark at room temperature. The percentage cytotoxicity was calculated with the following equation, using the luminescence signal: Percentage of cell death = 100 × ((sample‐blank)/(positive control‐blank)). The mixture of 5% FBS‐supplemented medium and AK detection reagent reaction buffer was used as the blank.

### 2.11 PDH enzyme activity assay

A kit from BioVision (K679‐100, Milpitas, CA, USA) was utilized to measure PDH activity according to the manufacturer's instructions.

### 2.12 AMPK *in vitro* kinase assay

Three kinds of AMPKα protein sources were used in the *in vitro* kinase assay. (a) AMPKα1‐containing proteins: the proteins immunoprecipitated by AMPKα1 antibodies; (b) AMPKα1/β1/γ1 human recombinant proteins (BML‐SE491, Enzo Life Science, Farmingdale, NY, USA); and (c) CaMKKβ‐pretreated AMPKα1/β1/γ1 complexes: the recombinant AMPKα1/β1/γ1 complexes (PKSH030314, Elabscience, Houston, TX, USA) were incubated with CAMKKβ recombinant proteins (PV4206, Thermo Fisher Scientific) at 30 °C for 30 min in 1× kinase buffer (#9802, Cell Signaling Technology) containing 200 μm ATP (#9804, Cell Signaling Technology) to yield phosphorylated/activated AMPK complexes (later as p‐AMPK complexes). The ratio of AMPKα1/β1/γ1 conc. versus CAMKKβ conc. was 4 : 1. The indicated 10‐fold dosages of compounds were incubated with 0.2 ng AMPKα‐related recombinant proteins or 25 μg AMPKα1‐containing proteins in the kinase reaction buffer (kinase buffer with 5 μm ATP and 10 mm DTT, #CY‐1182, MBL International, Woburn, MA, USA) at 30 °C for 30 min. After that, CycLex AMPK Kinase Assay Kit (#CY‐1182, MBL International) was performed according to the manufacturer’s instructions.

### 2.13 Quantitative PCR (qPCR)

qPCR was performed as described by Ponchel et al. [25]. Oligonucleotide sequences were as follows: HIF1α, 5′‐ATCCATGTGACCATGAGGAAATG‐3′ (forward) and 5′‐TCGGCTAGTTAGGGTACACTTC‐3′ (reverse); GAPDH, 5′‐CGACCACTTTGTCAAGCTCA‐3′ (forward) and 5′‐AGGGGTCTACATGGCAACTG‐3′ (reverse). Thermocycling was performed with a Roche LightCycler 480 sequence detection system (Roche Applied Science, Foster City, CA, USA). Expression levels of genes of interest were normalized to that of GAPDH in the same sample.

### 2.14 Immunoprecipitation and western blotting

The treated cells were lysed in ice‐cold Pierce IP Lysis buffer (87788, Thermo Fisher Scientific) supplemented with a protease and phosphatase inhibitor cocktail (78442, Thermo Fisher Scientific). The 400 μg whole‐cell extracts were incubated with the 4 μg indicated antibodies overnight at 4 °C with gentle rotation. The 20 μL protein A/G mix magnetic beads (LSKMAGAG02, Millipore, Darmstadt, Germany) were then added to the samples with gentle rotation for 4 h at 4 °C. The beads were washed with IP Lysis buffer four times and then boiled in sodium dodecyl sulfate loading buffer. For western blotting, cell extracts were separated by sodium dodecyl sulfate–polyacrylamide gel electrophoresis. After transferring the proteins, membranes were blocked and probed with antibodies. Bands were detected with an enhanced chemiluminescence system (GTX14698, Bioshop Canada, Burlington, ON, Canada). Western blotting was performed at least three times, and representative experiments are shown. Quantification was carried out using imagej software (NIH, Bethesda, MD, USA).

### 2.15 Metabolite measurements

PLC5 cells were seeded at a density of 1.5 × 10^6^ cells in 100‐mm dishes. After 24 h incubation, cells were treated with SCT‐1015 (20 μm) or DMSO in 5% FBS‐supplemented medium for 24 h. The treated cells were washed twice by using 5% (w/w) mannitol solution and were then incubated with methanol and a diluted internal standard solution (H3304‐1002, Human Metabolome Technologies (HMT), Yamagata, Japan) at room temperature for 30 s. The extracted solutions were transferred into microtubes and centrifuged at 2300 × *g*, 4 °C for 5 min. The supernatant was transferred to a Millipore 5‐kDa cutoff filter (UltrafreeMC‐PLHCC, HMT) and was centrifuged at 9100 × *g*, 4 °C for 3 h. The extracted sample solutions were used for C‐SCOPE‐selected component analysis at HMT. Targeted quantitative analysis was performed using capillary electrophoresis mass spectrometry (CE‐TOFMS and CE‐QqQMS) in the cation and anion analysis modes for analyzing cationic and anionic metabolites, respectively. Total protein abundance was used as the normalization reference, and the *P*‐value was computed by HMT using Welch's *t*‐test.

### 2.16 Measurement of glycolytic capacity

The real‐time extracellular acidification rate (ECAR) was measured using a Seahorse XF^e^24 Extracellular Flux Analyzer (Seahorse Bioscience, North Billerica, MA, USA) according to the manufacturer's instructions. PLC5 or Huh7 cells were seeded at a density of 1.5 × 10^6^ cells in 100‐mm dishes. After 24 h incubation, cells were treated with the indicated dose of SCT‐1015 or DMSO in 5% FBS medium for 24 h. The treated cells (7 × 10^4^·well^−1^ PLC5 or 4.2 × 10^5^·well^−1^) were reseeded with XF basal medium (2% FBS, 1% antibiotic‐antimycotic solution, 1% sodium pyruvate, and 4 mm L‐glutamine) in the Cell‐Tak (Corning, Corning, NY, USA) precoated Seahorse plate. After 30 min for cell attachment, the cells were incubated in a CO_2_‐free incubator for a further 30 min. ECAR (mpH·min^−^^1^) was then measured over time following the injection of glucose, oligomycin, and 2‐deoxyglucose (2‐DG).

### 2.17 Animal studies

PLC5, PLC5/shNT, or PLC5/shAMPKα1 (1 × 10^6^) cells were suspended in 100 μL of 1× PBS and then injected subcutaneously into 5‐week‐old male BALB/cAnN. Cg‐*Foxn1^nu^*/CrlNarl mice (obtained from the National Laboratory Animal Center, Taiwan). Huh7 (1 × 10^7^) cells were suspended in 200 μL of 1× PBS containing 50% Matrigel (354248, Corning) and then injected subcutaneously into 5‐week‐old male BALB/cAnN. Cg‐*Foxn1^nu^*/CrlNarl mice. When tumors reached 100 mm^3^, mice received vehicle (propylene glycol/Kolliphor HS 15/H_2_O), SCT‐1015 (25 mg·kg^−1^), metformin (250 mg·kg^−1^), rapamycin (2 mg·kg^−1^), or the combination of 2 mg·kg^−1^ rapamycin and 25 mg·kg^−1^ SCT‐1015 by mouth once daily. The tumor formation and growth sizes were measured with digital calipers. Tumor volumes were estimated using the formula for an ellipse, 0.523 × (short dimension)^2^ × (long dimension). Tumor samples were paraffin‐embedded, sectioned, and stained with specific antibodies for IHC analysis and examined by light microscopy. All animal experiments were performed in accordance with the Institutional Animal Care and Use Committee of National Yang Ming University (now as National Yang Ming Chiao Tung University). All experimental protocols involving mice were approved by the Ethical Committee of National Yang Ming University and performed in accordance with approved guidelines and regulations. Mice were housed in a rectangular mouse cage (area: 544 cm^2^, height: 14 cm) and were kept in a specific pathogen‐free environment under standard experimental conditions (light–dark cycle: 12 h, temperature: 20–22 °C, humidity: 50–70%). Five mice were housed in one cage and were cared for every day.

### 2.18 Immunohistochemistry (IHC)

The HCC patient tissue array was purchased from SuperBioChips Laboratories (SuperBioChips Laboratories, Seoul, Korea). IHC staining of the HCC patient tissue array was performed, using the VentanaBenchMark XT automated stainer (Ventana, Tucson, AZ). The slides were incubated with the HIF1α (1 : 50) and p‐AMPKα (1 : 25) primary antibody for 24 or 32 min at 37 °C, respectively, and then overnight at 4 °C. After three rinses in buffer, the slides were incubated with the secondary antibodies (EnVision System, DakoCytomation, Santa Clara, CA, USA). Tissue staining was visualized with a DAB substrate chromogen solution (DakoCytomation). Slides were then counterstained with hematoxylin, dehydrated, and mounted. Each run included, for each patient, PBS used as the primary antibody for the negative controls, while samples known to express these markers strongly served as the positive controls. This study was approved by the Ethics Committee of the Institutional Review Board of National Yang Ming University. Animal tumors were sectioned and then stained with the indicated antibodies (1 : 100) overnight at 4 °C. The IHC staining of animal tumors was then performed according to the manufacturer’s instructions of the LSAB2 streptavidin‐biotin complex system (Dako, Carpinteria, CA, USA).

### 2.19 Cyto‐ID autophagy detection assay

The 2.5 × 10^3^ cells·well^−1^ in 5% FBS supplemented medium were seeded into 96‐well plates with a black wall and a clear bottom. After 24 h incubation, the indicated dosages of SCT‐1015 or DMSO were treated for 48 h. After treatment, cells were stained with Cyto‐ID Green dye and Hoechst 33342 Nuclear Stain dye from the CYTO‐ID Autophagy Detection Kit (ENZ‐51031, Enzo Life Sciences, Farmingdale, NY, USA) for 30 min at 37 °C followed by washing with 1× assay buffer containing 5% FBS. The fluorescent signal was measured with Spark 10 m multimode microplate reader (Tecan, Männedorf, Switzerland). The CYTO‐ID Green and Hoechst 33342 Nuclear Stain can be read with an FITC filter (Excitation ~ 480 nm, Emission ~ 530) and DAPI filter set (Excitation ~ 340, Emission ~ 480), respectively. Cyto‐ID Green signal was normalized by Hoechst 33342 Nuclear Stain signal for cell number.

### 2.20 Statistical analysis

Data are presented as mean ± SD or mean ± SEM for *in vitro* and *in vivo* studies. graphpad Prism (GraphPad Software, La Jolla, CA, USA; Version 6) was used for analyzing the significance. *P < 0.05, **P < 0.01.

## 3 Results

### 3.1 SCT‐1015 is a novel direct AMPK activator

**Fig. 1:**
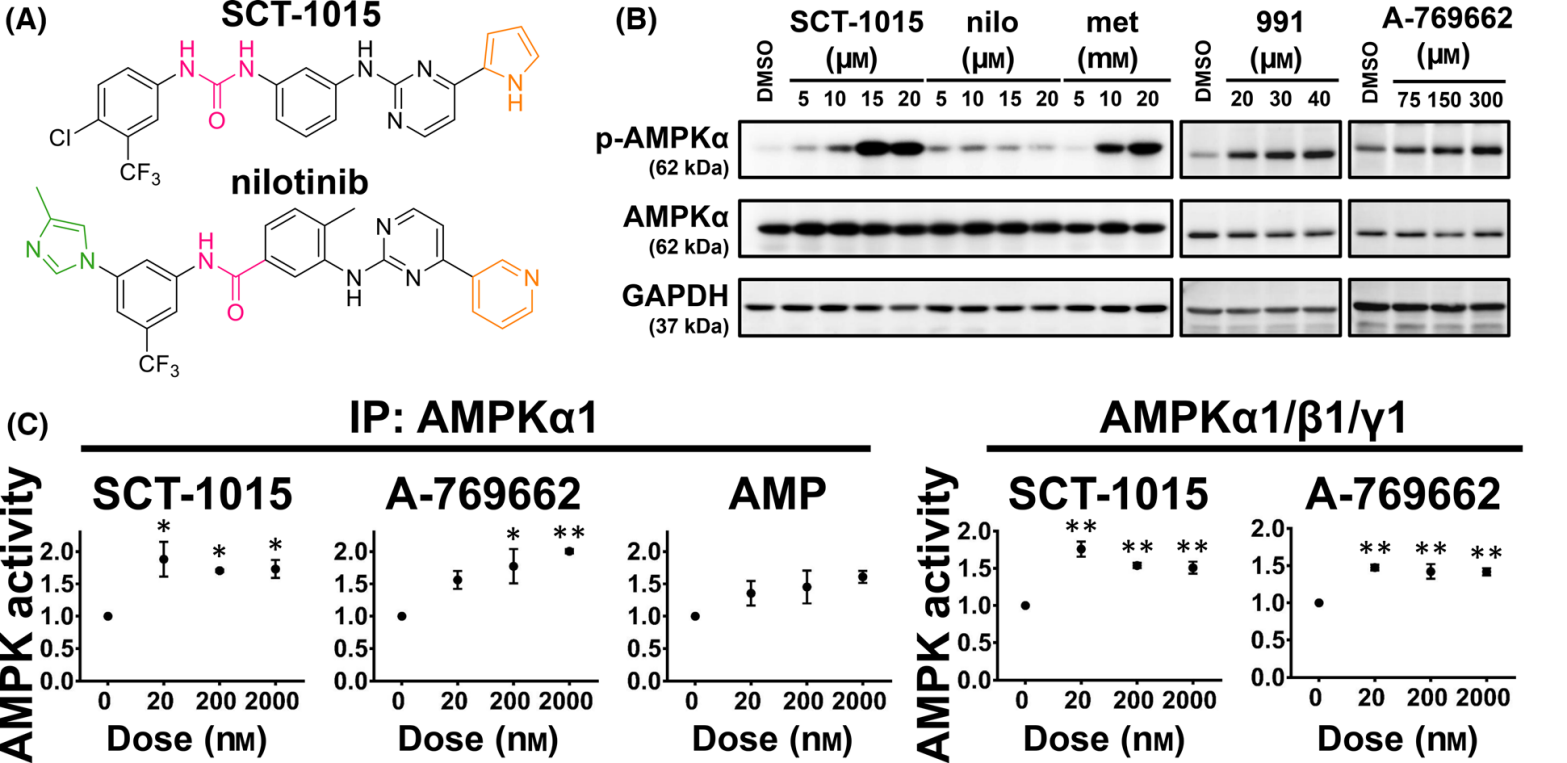
SCT‐1015 increased AMPK activity by direct interaction. (A) The chemical structures of SCT‐1015 and the lead compound (nilotinib). (B) PLC5 cells were exposed to SCT‐1015, nilotinib (nilo), metformin (met), 991, or A‐769662 in a serial dosage for 24 h, and the protein abundance of p‐AMPKα, AMPKα, and GAPDH (control) were analyzed by western blotting. The 0.1% DMSO was used as vehicle control. The groups for western blotting were performed in three independent replicates. (C) SCT‐1015 showed a direct effect on AMPK activity. AMPKα1‐containing lysates were collected by AMPKα1 Ab and incubated with SCT‐1015, A‐769662, and AMP at the indicated dose for 30 min *in vitro* (*left*). Recombinant AMPKα1/β1/γ1 protein was coincubated with SCT‐1015 or A‐769662 at the indicated dose for 30 min *in vitro* (*right*). Symbols, mean; error bars, SEM (*n* = 3); *P < 0.05, **P < 0.01 (one‐way analysis of variance [ANOVA] with Tukey’s HSD test).

AMPK activation exerts the suppressive function during HCC progression. Nilotinib has been proven to be an AMPK activator through PP2A inhibition in HCC [21]. In addition, nilotinib can also target bcr‐abl for the treatment of chronic myelogenous leukemia [26]. To further enhance the AMPK activation effect of nilotinib, we used it as a lead compound to synthesize a novel potential AMPK activator, SCT‐1015 (Fig. 1A). In the design of SCT‐1015, we aimed to strengthen the specificity of AMPK activation to avoid the effect on abl. We modified the functional groups to abolish the abl kinase‐binding affinity. The pyridyl ring was replaced by a pyrrole to diminish the hydrogen bond formation with Met318 in the hinge region of abl kinase, and the imidazole ring was removed to reduce the van der Waals interactions. Such modifications would affect the overall structural characteristics; thus, the linker from amide was then replaced by a longer urea to maintain a similar structure size and length of SCT‐1015 for the preservation of the potential of AMPK activation.

**Fig. 2:**
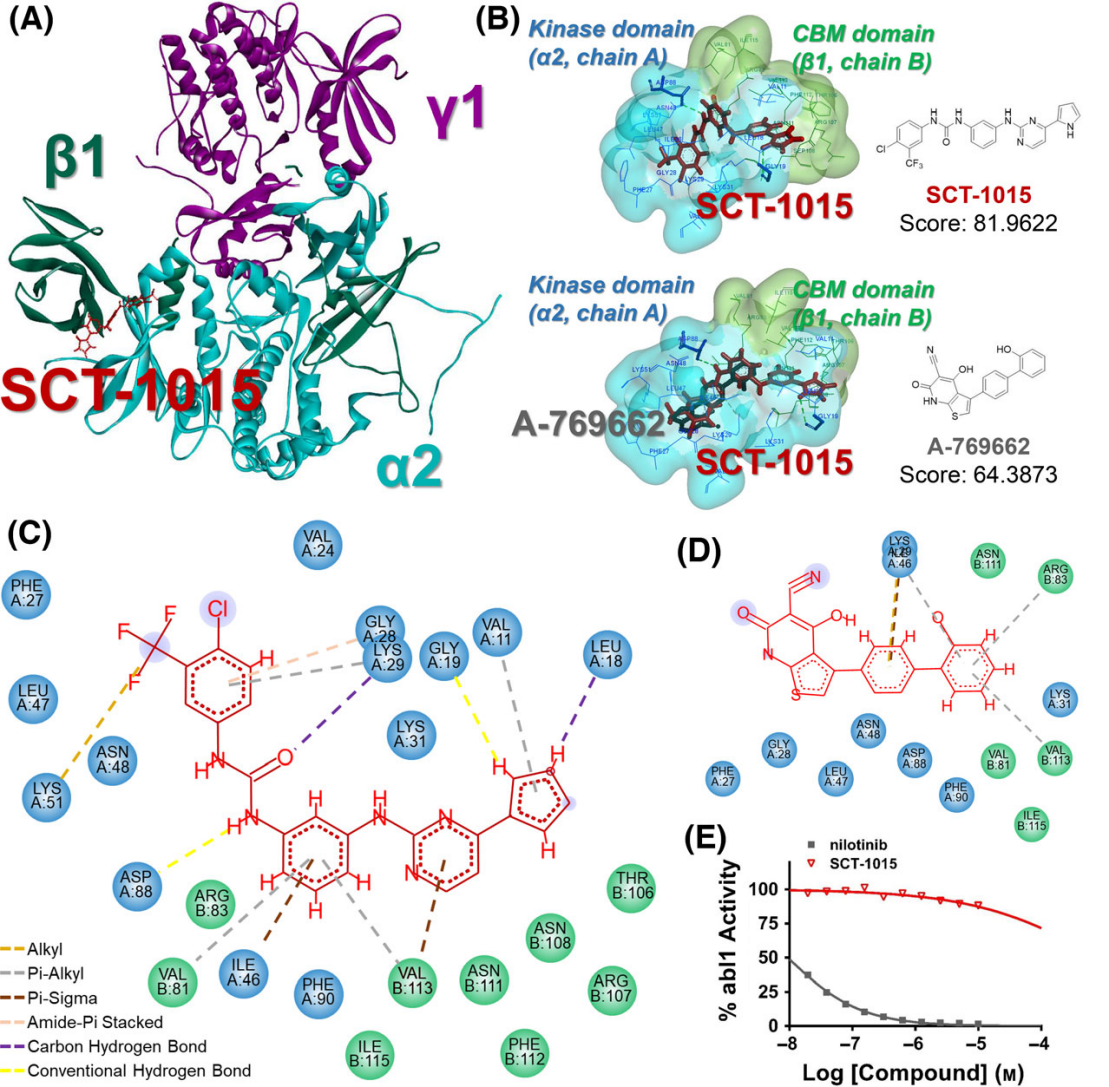
The binding mode of SCT‐1015 and AMPK. (A) The 3D structure of full‐length α2β1γ1 in complex with the SCT‐1015 (red sticks). Cyan: α2‐subunit, dark green: β1‐subunit, purple: γ1‐subunit. (B) *Upper,* the binding view of SCT‐1015 in the pocket at the interface between the CBM (green) and the kinase domain (blue). *Bottom*, the overlay of A‐769662 (gray) and SCT‐1015 (red) is shown in the stick representation. Score, GOLD fitness score. (C,D) 2D interaction diagrams of SCT‐1015 (C) or A‐769662 (D) with the CBM domain (β1, chain B) and the kinase domain (α2, chain A) (E) Ten doses of nilotinib or SCT‐1015 were tested in Abl1 kinase assay with 2‐fold serial dilution starting at 10 μμm. Symbols, mean (*n* = 2).

We then compared the AMPK‐activating ability between SCT‐1015 and nilotinib in HCC using PLC5 cells. AMPK activation can be examined by its phosphorylation level. The phosphorylation on Thr172 of the AMPKα subunit was increased in a dose‐dependent manner under SCT‐1015 treatment. Such phosphorylation was drastically stronger compared with the treatment using nilotinib and was also stronger than the currently available AMPK activators, including metformin, A‐769662, and 991 (Fig. 1B). Since the induced phosphorylation level by SCT‐1015 was much higher than that of its lead compound, we suspected that SCT‐1015 may target AMPK directly. We then performed four experimental assays to test this hypothesis. First, we treated SCT‐1015 to the endogenous AMPKα1 pulled down from PLC5 cells or to the recombinant AMPKα1/β1/γ1 complexes, and an *in vitro* kinase assay was used to quantify the AMPK activity. The endogenous AMPKα1 and the recombinant complexes were both activated at very low SCT‐1015 concentrations (nm) (Fig. 1C). The known AMPK direct activators, AMP and A‐769662, were used as positive controls (Fig. 1C). Second, in the CaMKKβ‐pretreated AMPKα1/β1/γ1 complexes (later as p‐AMPK complexes), SCT‐1015 alone treatment also increased the p‐AMPK complex activity (Fig. S3). In addition, p‐AMPK complexes activity was further enhanced by the combination of SCT‐1015 and AMP, which suggested the distinct interaction sites of SCT‐1015 and AMP (γ‐subunits) (Fig. S3). Third, molecular docking was further performed to analyze the potential binding patterns of SCT‐1015 to AMPK. SCT‐1015 was inserted into the pocket of the CBM domain of AMPKβ and the kinase domain of AMPKα (Fig. 2A,B, *upper*). We found that the binding pattern of SCT‐1015 to AMPK was similar to A‐769662 [22] (Fig. 2B, *bottom*). Compared with A‐769662, SCT‐1015 showed a higher binding score to AMPK (SCT‐1015: 81.9622; A‐769662: 64.3873). Fourth, from the 2D interaction diagram, such a higher binding score was caused by the possible π‐sigma interaction, π‐alkyl interaction, conventional hydrogen bond, and carbon‐hydrogen bond in the binding pocket of AMPK (Fig. 2B, *bottom*, 2C,D). From the results of two *in vitro* kinase assays, the molecular docking analysis, and the 2D interaction diagram, we demonstrated the direct binding between SCT‐1015 and AMPK.

Since nilotinib activated AMPK through PP2A inhibition [21], we performed the PP2A activity assay to elucidate whether SCT‐1015 showed a similar ability on PP2A inhibition for AMPK activation. In PLC5 cells, SCT‐1015 did not suppress PP2A activity (Fig. S4). The known PP2A activator, D‐erythro‐Sphingosine (DES), was used as a positive control (Fig. S4). In addition to the enhanced AMPK activation, SCT‐1015 was also designed to avoid the direct binding to abl kinase. The kinase assay showed that the abl1 kinase activity was not affected by SCT‐1015 treatment (Fig. 2E), and the treatment of nilotinib was used as a positive control (Fig. 2E). These results were consistent with the purpose of our design for SCT‐1015 that the abl kinase targeting ability in nilotinib was completely removed to allow for a more specific and enhanced targeting effect of SCT‐1015 to AMPK.

### 3.2 SCT‐1015 inhibited HCC proliferation and elicited an anti‐Warburg effect

**Fig. 3:**
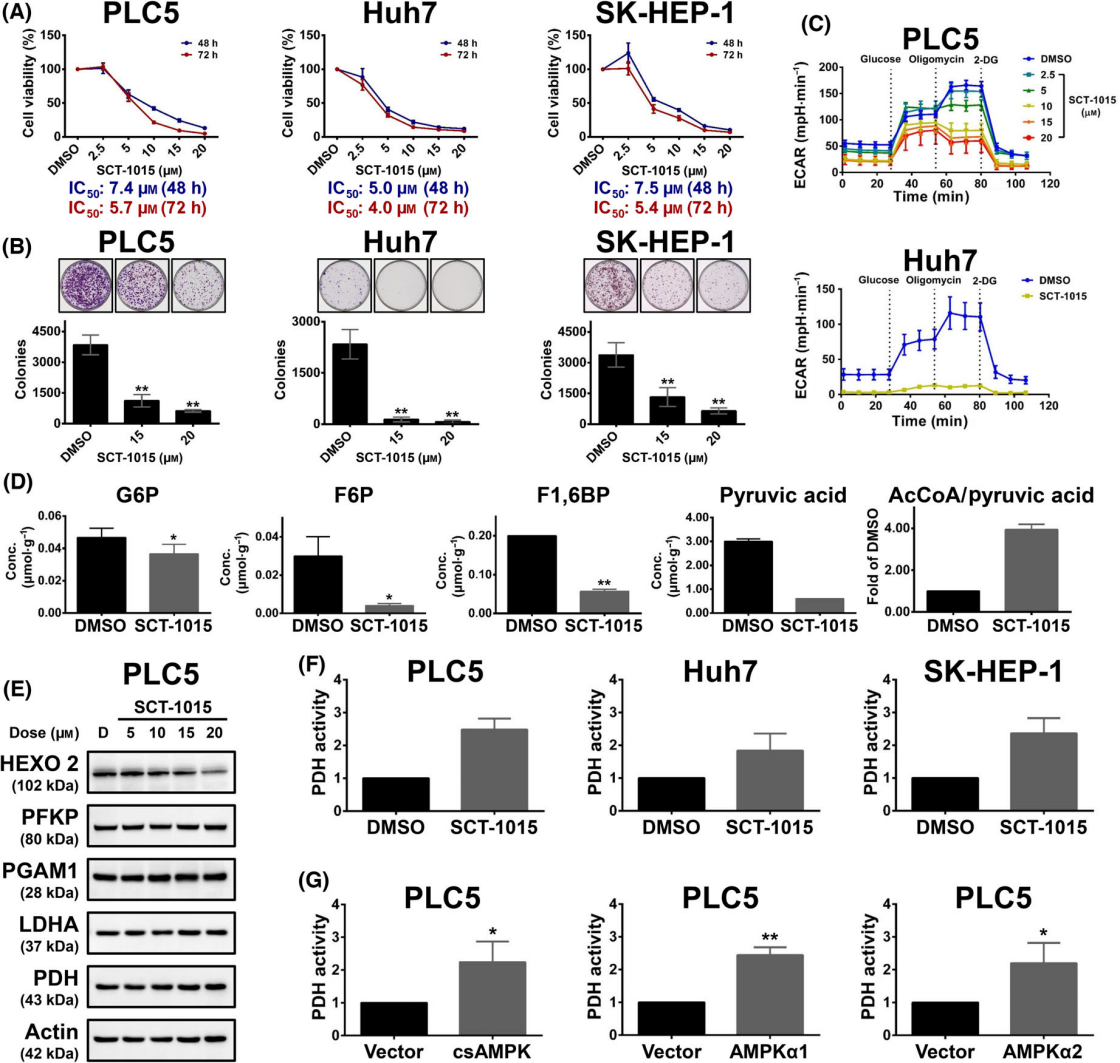
SCT‐1015 remodeled metabolic status and impaired cell survival in HCC. (A) Three HCC cells (PLC5, Huh7, or SK‐HEP‐1) were exposed to SCT‐1015 at the indicated doses for 48 or 72 h. The cell viability was assessed by PrestoBlue™ cell viability reagent. The values of IC_50_ were calculated by nonlinear regression using log (inhibitor) vs. normalized response‐variable slope model (graphpad Prism software). Symbols, mean; error bars, SD (*n* = 3). (B) Three HCC cells (PLC5, Huh7, or SK‐HEP‐1) were exposed to SCT‐1015 at the indicated doses for 48 h. The colony‐forming ability was analyzed by colony formation assay. Columns, mean; error bars, SD (*n* = 3); **P < 0.01 (one‐way ANOVA with Tukey’s HSD test). (C) Real‐time monitored glycolytic activity was determined by measuring the ECAR using the Seahorse XF^e^24 Extracellular Flux Analyzer. PLC5 (*upper*) or Huh7 (*bottom*) cells were treated with SCT‐1015 at the indicated doses for 24 h. ECAR (mpH·min^−1^) was measured over time following the injection of glucose (10 mm), oligomycin (1 μμm), and 2‐DG (50 mm). The values are shown as the mean ± SD (*n* = 3). (D) The change of the metabolites (glucose 6‐phosphate, G6P; fructose 6‐phosphate, F6P; fructose 1,6‐bisphosphate, F1,6BP; pyruvic acid) and the ratio of AcCoA/Pyruvic acid in SCT‐1015‐treated PLC5 cells were analyzed. Targeted quantitative analysis was performed using capillary electrophoresis mass spectrometry (CE‐TOFMS or CE‐QqQMS). Columns, mean; error bars, SD (*n* = 3); *P < 0.05, **P < 0.01 (Welch’s *t*‐test). (E) Dose‐dependent response of SCT‐1015 on glycolysis‐related proteins. PLC5 cells were treated with different concentrations of SCT‐1015 for 24 h. The indicated proteins (hexokinase 2, HEXO 2; platelet‐type phosphofructokinase, PFKP; phosphoglycerate mutase, PGAM1; lactate dehydrogenase A, LDHA; pyruvate dehydrogenase, PDH; Actin) were determined by western blotting. The groups for western blotting were performed in three independent replicates. (F) HCC cells were treated with SCT‐1015 (20 μμm) for 24 h, and the PDH activity was analyzed by a PDH activity assay. Columns, mean; error bars, SD (*n* = 3). (G) PLC5 cells were transfected with corresponding vectors, csAMPK, AMPKα1, or AMPKα2 for 72 h, and the PDH activity was analyzed. Columns, mean; error bars, SD (*n* = 3); *P < 0.05, **P < 0.01 (Student’s *t‐*test, two‐tailed).

We further tested the antitumor ability of SCT‐1015 in several human HCC cell lines, the PLC5, SK‐HEP‐1, and Huh7 cells. SCT‐1015 decreased the cell viability in both dose‐ and time‐dependent manners (Fig. 3A), impaired the clonogenic capacity of cells (Fig. 3B), and induced cell death (Fig. S5). Since AMPK was found to negatively regulate the Warburg effect in lymphomagenesis [7], we then examined whether the metabolic pathways were affected in HCC under SCT‐1015 treatment due to the AMPK activation. The extracellular acidification rate (ECAR) was used to reveal the lactate production of aerobic glycolysis. SCT‐1015 remarkably reduced the basal and glycolytic capacity in a dose‐dependent manner in PLC5 cells (Fig. 3C, *upper*). A similar ECAR reduction was also observed in Huh7 cells (Fig. 3C, *bottom*). We then tested the major metabolites along the glycolysis pathway from glucose to pyruvate then to acetyl‐CoA. Capillary electrophoresis mass spectrometry (CE‐MS) was used to measure the absolute concentration of these major metabolites in PLC5 cells under SCT‐1015 treatment. The metabolite concentration was relatively low in the SCT‐1015‐treated group, including glucose 6‐phosphate, fructose 6‐phosphate, fructose 1,6‐bisphosphate, and pyruvic acid. The ratio of AcCoA/pyruvic acid, the initial flux of OXPHOS, increased in the SCT‐1015‐treated group (Fig. 3D). From Fig. 3C to Fig. 3D, these results suggested that SCT‐1015 can shift the metabolic pathway from aerobic glycolysis (dominant in tumor cells) towards OXPHOS (dominant in normal cells). The decreased cellular ATP levels were observed under SCT‐1015 treatment (Fig. S6), which may be due to the weakened glycolysis flux of SCT‐1015.

**Fig. 4:**
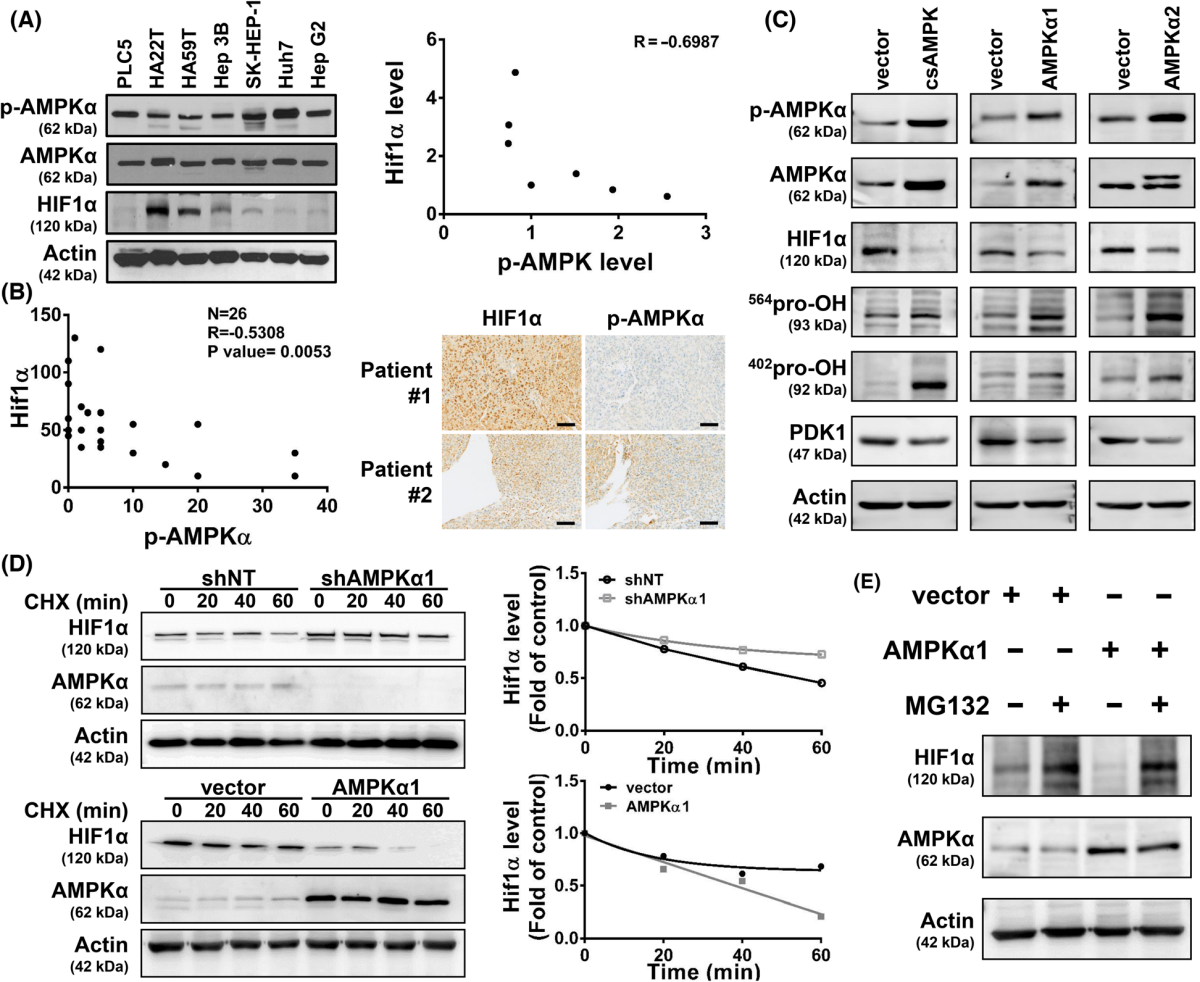
p‐AMPK was negatively correlated with HIF1α in HCC. (A) *Left*, Protein expression pattern of p‐AMPKα, AMPKα, HIF1α, and Actin in seven HCC cell lines were analyzed by western blotting. The groups for western blotting were performed in three independent replicates. *Right*, The correlation of p‐AMPKα and HIF1α in seven HCC cell lines (Pearson’s *r* = −0.6987). (B) *Left*, p‐AMPKα and HIF1α were stained in tumor tissues of 26 patients with HCC. Pearson’s *r* was −0.5308 (*P* = 0.0053). *Right*, the IHC staining from two patients (patients #1 and #2) is shown (the scale bars represent 100 μm). (C) PLC5 cells were transfected with corresponding vectors, csAMPK, AMPKα1, or AMPKα2 for 72 h, and the indicated proteins (p‐AMPKα; AMPKα; HIF1α; ^564^pro‐OH HIF1α, ^564^pro‐OH; ^402^pro‐OH HIF1α, ^402^pro‐OH; pyruvate dehydrogenase kinase 1, PDK1; Actin) were analyzed by western blotting. The groups for western blotting were performed in three independent replicates. (D) PLC5 cells were transfected with corresponding vectors, AMPKα1, or shAMPKα1, for 72 h, and then cycloheximide (CHX, 50 μg·mL^−1^) was exposed at the indicated times. HIF1α, AMPKα, and Actin were analyzed by western blot (*left*). The groups for western blotting were performed in three independent replicates. After normalizing with the loading control (Actin), the protein abundance of HIF1α is shown, *right*. (E) PLC5 cells were transfected with the corresponding vector and AMPKα1 for 72 h and exposed with or without the proteasome inhibitor MG132 (100 μm) for a further 4 h. The protein abundance of HIF1α, AMPKα, and Actin was analyzed by western blotting. The groups for western blotting were performed in three independent replicates.

**Fig. 5:**
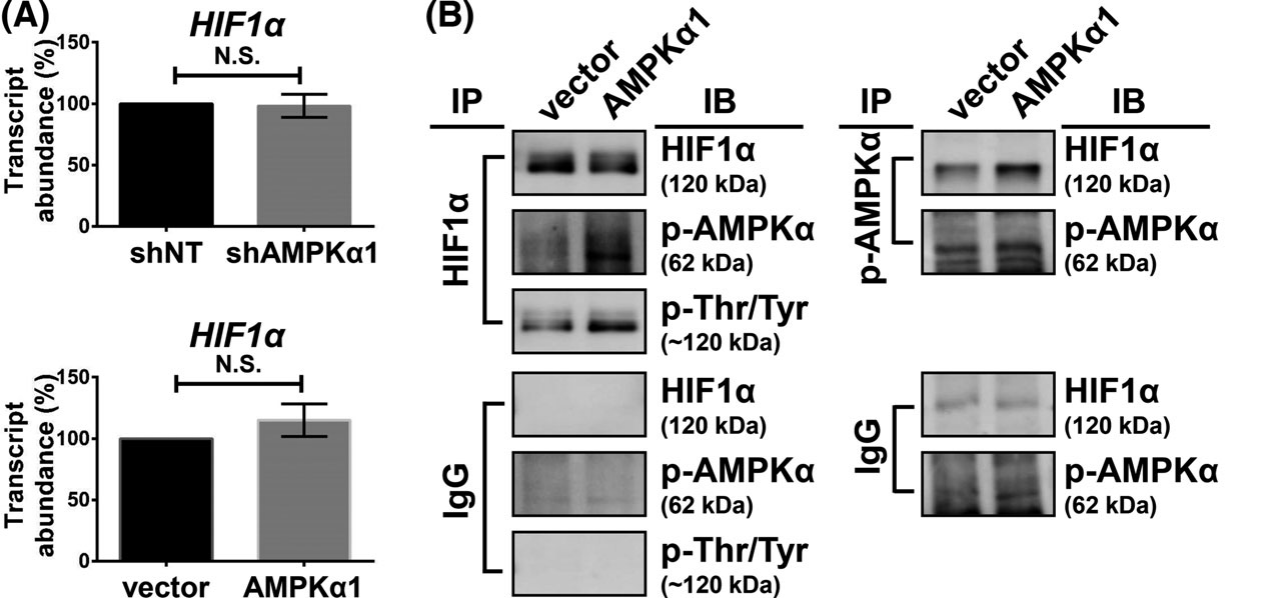
Transcriptional and post‐translational analysis of HIF1α. (A) PLC5 cells were transfected with corresponding vectors, AMPKα1, or shAMPKα1 for 72 h, and the *HIF1A* expression was analyzed by qPCR. Columns, mean; error bars, SD (*n* = 3), N.S., not significant (Welch’s *t*‐test). (B) PLC5 cells were transfected with the corresponding vector and AMPKα1 for 72 h. Cell lysates were collected for the immunoprecipitation assay. IgG antibody was used as a negative control of immunoprecipitation assay. The experiments were performed in three independent replicates.

**Fig. 6:**
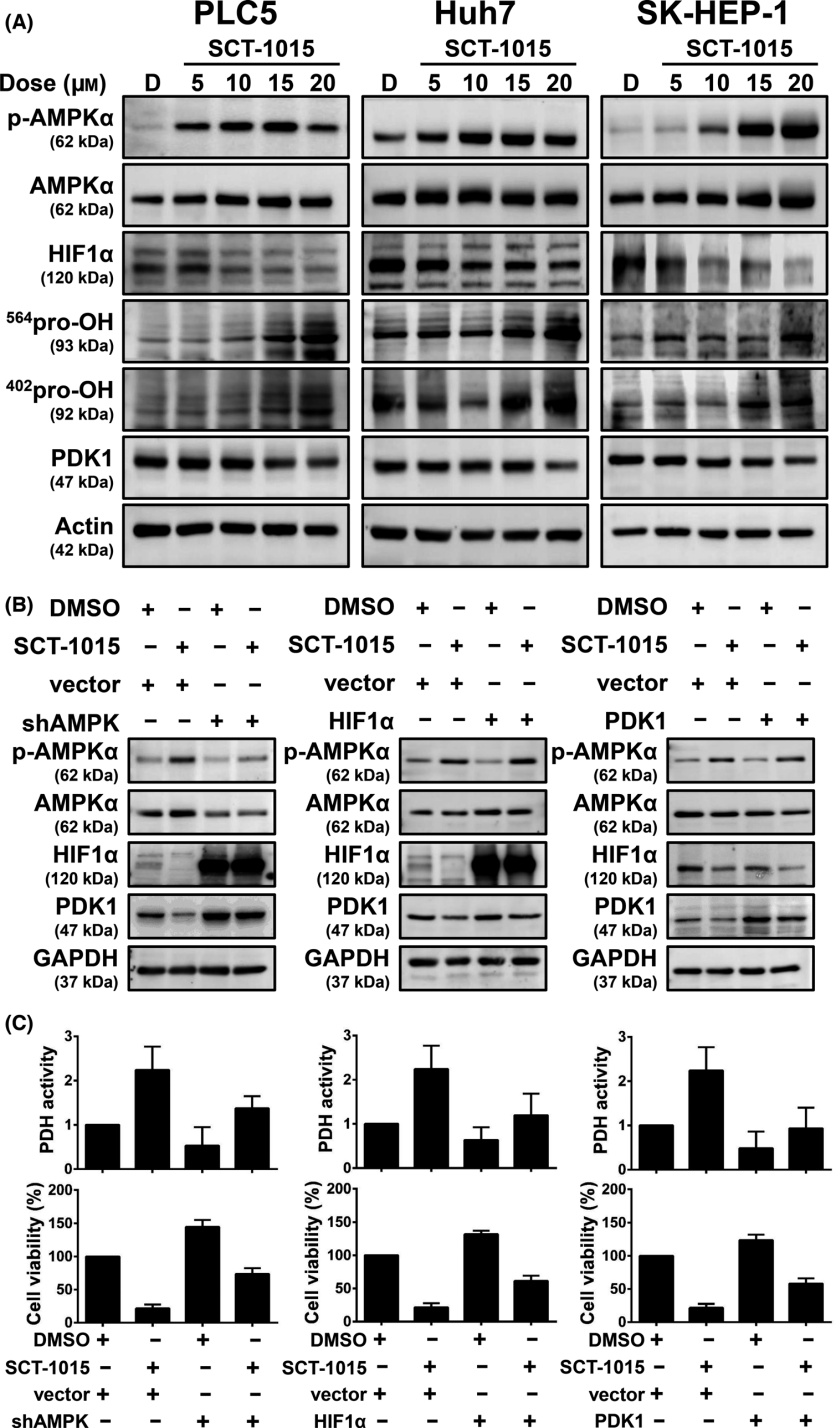
SCT‐1015 triggered the AMPK/HIF1/PDK1 axis. (A) Protein abundance of p‐AMPKα, AMPKα, HIF1α, ^564^pro‐OH, ^402^pro‐OH, and PDK1 were analyzed by western blotting after SCT‐1015 treatment for 24 h. The groups for western blotting were performed in three independent replicates. (B) PLC5 cells were transfected with the corresponding vector, shAMPKα1 (*left*), HIF1α overexpression plasmid (*middle*), or PDK1 overexpression plasmid (*right*). After 72 h transfection, cells were treated with SCT‐1015 (20 μm) for 24 h and subjected to western blotting assay. The groups for western blotting were performed in three independent replicates. (C) PLC5 cells were transfected with the corresponding vector, shAMPKα1 (*left*), HIF1α overexpression plasmid (*middle*), or PDK1 overexpression plasmid (*right*). After 72 h transfection, cells were treated with SCT‐1015 (20 μm, 48 h) for viability analysis (*bottom*, columns, mean; error bars, SD; *n* = 3) or exposed to SCT‐1015 (20 μm, 24 h) for PDH activity analysis (*upper*, columns, mean; error bars, SD; *n* = 3).

We continued to examine the protein abundance of the glycolysis‐ and OXPHOS‐related enzymes affected by SCT‐1015. The hexokinase 2 was reduced (Fig. 3E), which is consistent with the weakened aerobic glycolysis [27]. Pyruvate dehydrogenase kinase 1 was also decreased (Fig. 6A), which may lead to less phosphorylation of pyruvate dehydrogenase. Without phosphorylation, pyruvate dehydrogenase would become activated to transfer pyruvate to acetyl‐CoA for entering OXPHOS. We then tested whether the increased pyruvate dehydrogenase activity is regulated by AMPK activation from the SCT‐1015 treatment (Fig. 3F). The constitutively active AMPKα1 was overexpressed in PLC5 cells, resulting in the enhanced pyruvate dehydrogenase (Fig. 3G). This regulation was also observed in AMPKα1‐ and AMPKα2‐overexpressed PLC5 cells (Fig. 3G). Our results demonstrated that SCT‐1015 affected the metabolic pathway in HCC through the AMPK activation to regulate the related enzyme protein abundance and activity to deteriorate the HCC cell viability and colony formation.

### 3.3 AMPK‐regulated HIF1α protein stability in HCC

Transcription factor HIF1α has been discovered to control several metabolic enzymes, such as hexokinase and pyruvate dehydrogenase kinase in numerous cancer types, which lead to the Warburg phenotype [28]. In normoxic conditions, the enhanced HIF1α protein stability has been observed in acute or chronic depletion of AMPKα [7, 29], but the relationship between AMPK and HIF1α remained unclear in HCC. We first examined p‐AMPKα, AMPKα, and HIF1α abundance in several human HCC cell lines, and the suppressed p‐AMPK abundance showed a negative correlation with the high HIF1α (*R* = −0.6987) (Fig. 4A). We then clinically investigated such correlations using IHC staining in 26 HCC patient specimens. The p‐AMPK was also inversely correlated with HIF1α (*R* = −0.5308) (Fig. 4B, *left*). Strong HIF1α staining correlated with low levels of p‐AMPKα in patient samples (Fig. 4B, *right*, *top*). Weak HIF1α staining showed high levels of p‐AMPKα protein (Fig. 4B, *right*, *bottom*). Both HCC cells and the clinical tissues showed significantly negative correlation between the p‐AMPKα and HIF1α abundance. We then investigated whether HIF1α abundance was regulated by p‐AMPKα. The HIF1α protein level was reduced in the PLC5 cells overexpressing constitutively active AMPKα1 (p‐AMPKα). Similar results were observed in the AMPKα1 and AMPKα2 overexpression cells because the p‐AMPKα levels increased due to the induced AMPK protein abundance (Fig. 4C). The abundance of a protein could be regulated by its synthesis or degradation rate. We generated AMPKα1‐knockdown cells for the comparison with the AMPK‐overexpressing cells to elucidate the HIF1α protein synthesis and degradation. A protein synthesis inhibitor, cycloheximide (CHX), was treated to the cells followed by a time‐course analysis to analyze the HIF1α degradation. Silencing AMPK suppressed HIF1α degradation, while overexpressing of AMPK accelerated the degradation (Fig. 4D). The normoxic HIF1α protein stability is downregulated by proline hydroxylation through the ubiquitin–proteasome system, which is the major post‐translational regulation of HIF1α half‐life. Proline hydroxylation (564P and 402P) was increased in the AMPK‐overexpressing cells, which is consistent with the lower protein abundance of HIF1α (Fig. 4C). A proteasome inhibitor, MG132, was further treated to the AMPK‐overexpressing cells, which enhanced the HIF1α stability (Fig. 4E). The transcript abundance of *HIF1α* was not affected in both AMPK‐knockdown and AMPK‐overexpressing cells, suggesting that *HIF1α* was regulated by AMPK through the protein level instead of the transcript level (Fig. 5A). To further examine the effect of AMPK to HIF1α on the protein level, we performed immunoprecipitation using HIF1α and p‐AMPK antibodies to test their interacting proteins. Both HIF1α and p‐AMPK could be reciprocally detected in the two immunoprecipitation experiments. The threonine/tyrosine phosphorylation of the 120 kDa proteins, as the molecular weight of HIF1α, was further induced under AMPK α1 overexpression (Fig. 5B). These results suggested that AMPK may increase HIF1α phosphorylation through a direct protein–protein interaction or indirect signaling transduction, which increased HIF1α hydroxylation for entering the ubiquitin–proteasome system.

### 3.4 SCT‐1015 regulated the AMPK/HIF1α‐mediated signaling pathway

As mentioned above, we found that SCT‐1015 enhanced pyruvate dehydrogenase activity (Fig. 3F) to increase the OXPHOS status and inhibited the cell viability in HCC (Fig. 3A). In HCC, we proved that AMPK induced HIF1α degradation and reduced pyruvate dehydrogenase kinase 1 protein abundance (Fig. 4). We therefore suspected whether SCT‐1015 regulated HIF1α and pyruvate dehydrogenase kinase 1 and subsequently led to OXPHOS flux change and retarded cell growth through AMPK activation in HCC. Dose‐ and time‐escalating SCT‐1015 treatments were conducted in HCC cells, and the protein abundance of HIF1α and pyruvate dehydrogenase kinase 1 was reduced, with a corresponding increase in Thr172 phosphorylation on the AMPKα (Fig. 6A). We further used the cells with transient perturbation of pyruvate dehydrogenase kinase or HIF1α overexpression or AMPKα1 silencing to test the effect of SCT‐1015 treatment. Silencing of AMPK rescued the decreased protein levels of HIF1α and pyruvate dehydrogenase kinase 1 (Fig. 6B, *left*). In HIF1α overexpression cells, the reduced pyruvate dehydrogenase kinase protein abundance was recovered (Fig. 6B, *middle*). Pyruvate dehydrogenase kinase overexpression counteracted the SCT‐1015 treatment on its own protein abundance (Fig. 6B, *right*). Pyruvate dehydrogenase activity and cell viability were analyzed to determine the contribution of the AMPK/HIF1α‐mediated cascade to the glycolytic phenotype and tumor cell progression induced by SCT‐1015. All three transient perturbed cells rescued the growth and inhibited the SCT‐1015‐enhanced pyruvate dehydrogenase activity (Fig. 6C). These results demonstrated that SCT‐1015 regulated the AMPK/HIF1α‐mediated signaling pathway to achieve the antitumor effects.

### 3.5 SCT‐1015 targeted both ERK and mTORC1 axes for the reduced HIF1α

SCT‐1015 increased the proline hydroxylation on HIF1α (Fig. 6A). The hydroxylation sites are on aa564 and aa402 proline, which were proved as the substrates of prolyl hydroxylases (PHD) [30]. Phosphorylation of PHD by extracellular regulated kinase (ERK) prevents the binding between PHD and HIF1α [31]. AMPK activators have been found to inhibit ERK signaling [32]. In other words, AMPK activators can promote the binding between PHD and HIF1α to facilitate the protein degradation of HIF1α. As an AMPK activator, SCT‐1015 may also inhibit ERK signaling to induce the protein degradation of HIF1α through proline hydroxylation. We first examined the ERK phosphorylation in three HCC cells (PLC5, Huh7, and SK‐HEP‐1) under the treatment of SCT‐1015 (Fig. S7A). SCT‐1015 could suppress ERK phosphorylation, which represented the inhibition of ERK activity. Immunoprecipitation assay was then performed for the validation of the interaction between HIF1α and PHD triggered by SCT‐1015. Compared with the DMSO control group, the reduced HIF1α was immunoprecipitated by HIF1α antibodies and was accompanied by a slight increase of immunoblotted PHD3 in the SCT‐1015 treatment group. The increased ratio of PHD3/HIF1α (1.6‐fold) revealed their enhanced interaction and the increase of subsequent HIF1α hydroxylation under SCT‐1015 treatment (Fig. S7B). We further applied the inhibitor of HIF prolyl hydroxylase, roxadustat [33], to elucidate whether the reduced HIF1α of SCT‐1015 was mediated through the hydroxylation of HIF1α. Roxadustat rescued the decreased protein levels of HIF1α under the treatment of SCT‐1015, suggesting that the hydroxylation of HIF1α of SCT‐1015 resulted in the reduced HIF1α (Fig. S7C). Besides the inhibition of ERK signaling for promoting the protein degradation of HIF1α. The mechanistic target of rapamycin complex 1 (mTORC1) has been found to control the protein translation of HIF1α through 4E‐BP1 and S6K phosphorylation [34]. For validation of the mTORC1 axis, we examined the 4E‐BP1 and S6K phosphorylation, which represented the mTORC1 activity [35], in three HCC cells (PLC5, Huh7, and SK‐HEP‐1) under the treatment of SCT‐1015 (Fig. S8A). SCT‐1015 reduced the phosphorylation of 4E‐BP1 and S6K in a dose‐dependent manner, indicating that mTORC1 activity was inhibited by SCT‐1015 (Fig. S8A). Since the inactivation of mTORC1 resulted in the induction of autophagy, we examined whether the autophagy flux was induced by SCT‐1015 using the Cyto‐ID autophagy detection assay. The autophagy flux was increased under SCT‐1015 treatment, which was likely related to the mTORC1 inactivation (Fig. S8B). These results indicated that both ERK and mTORC1 axes may contribute to the reduced protein level of HIF1α.

### 3.6 SCT‐1015 exerted antitumor effect *in vivo*

**Fig. 7:**
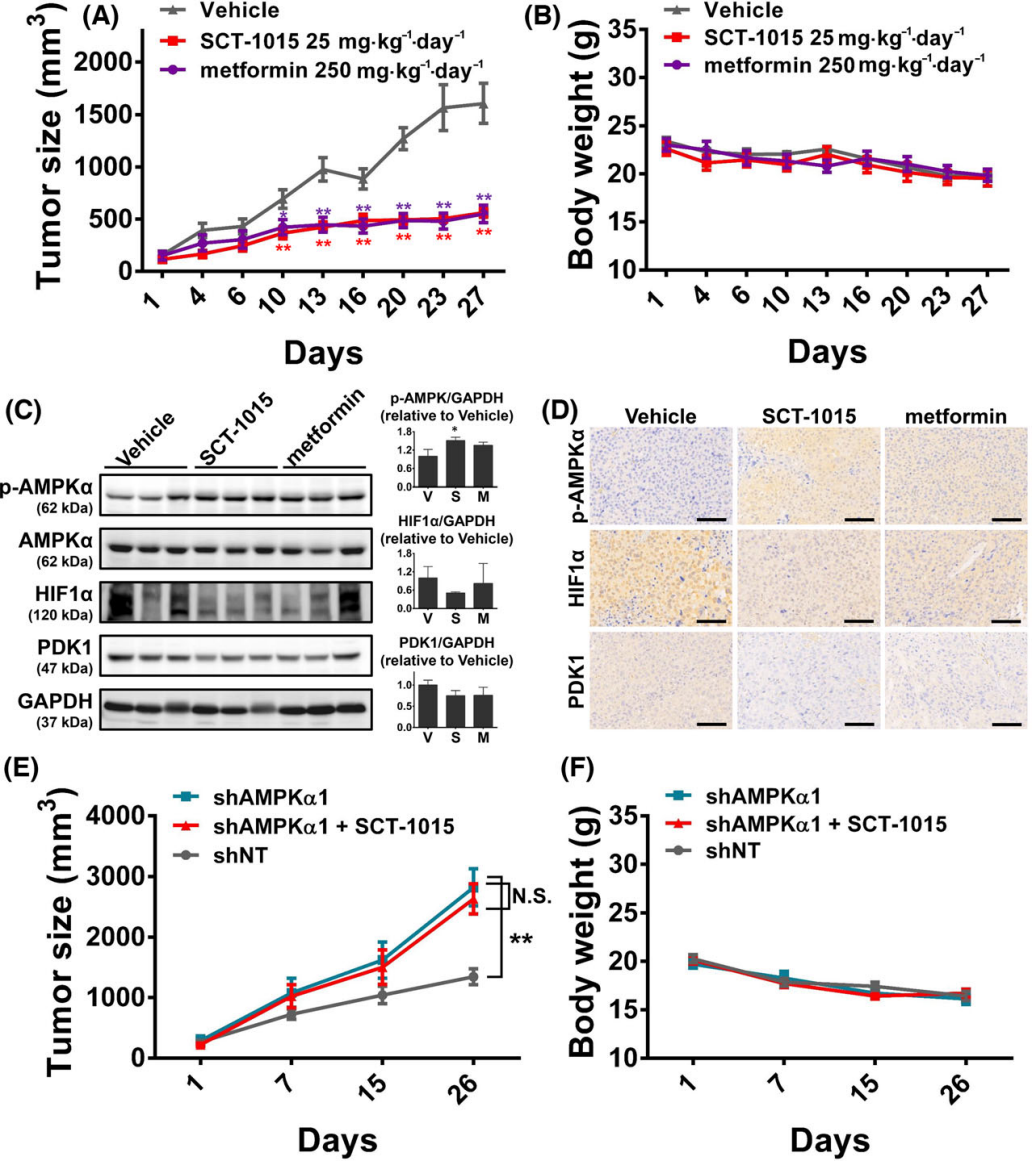
*In vivo* effects of SCT‐1015 on PLC5 xenograft mice model. (A,B) The tumor growth curve (A) and body weight (B) of PLC5‐bearing mice treated with vehicle, SCT‐1015 (25 mg·kg^−1^·five days per week^−1^·oral^−1^), or metformin (250 mg·kg^−1^·five days per week^−1^·oral^−1^). The values were shown as the mean ± SEM (*n* = 6). *P < 0.05, **P < 0.01 (one‐way ANOVA with Tukey’s HSD test). (C) *In vivo* protein levels of p‐AMPKα, AMPKα, HIF1α, PDK1, and GAPDH were analyzed by western blotting (*left*). The blots in (C, *left*) were quantified using imagej software, and the quantitative results are then shown (C, *right*). The protein abundance of GAPDH was used for the normalization of protein loading. Columns, mean; error bars, SD (*n* = 3 in each treatment group); **P < 0.01 (Student’s *t‐*test, two‐tailed). (D) IHC staining of the xenograft tumor sections with indicated treatment (*n* = 3 in each treatment group). The scale bars represent 100 μm. (E,F) Nude mice were subcutaneously injected with PLC5‐shNT or PLC5‐shAMPKα1 cells. Mice were treated with SCT‐1015 or vehicle for 26 days, and the tumor size (E) or body weight (F) was measured. Symbols, mean; error bars, SEM (*n* = 7); **P < 0.01; N.S., not significant (one‐way ANOVA with Tukey’s HSD test).

To determine the *in vivo* antitumoral properties and mechanisms of SCT‐1015, nude mice bearing PLC5 or Huh7 xenografts were used (Fig. 7 and Fig. S9). SCT‐1015 significantly attenuated the tumor progression (25 mg·kg^−1^·five days per week^−1^·oral^−1^ in PLC5‐bearing mice or 25 mg·kg^−1^·day^−1^·oral^−1^ in Huh7‐bearing mice) without an apparent effect on body weight or marked toxicity in comparison with the vehicle group (Fig. 7 and Fig. S9). The treatment dosage of SCT‐1015 was 10‐fold lower than another AMPK activator, metformin (250 mg·kg^−1^·five days per week^−1^·oral^−1^) with a similar tumor suppression tendency, indicating the strong potential in clinical therapeutic applications. Consistent with the *in vitro* results, SCT‐1015 increased the phosphorylation of AMPK and reduced the protein abundance of HIF1α and pyruvate dehydrogenase kinase 1. Such effects were much stronger than metformin (Fig. 7C,D). To investigate the role of AMPK in the SCT‐1015‐mediated antitumoral properties *in vivo*, we monitored the antitumoral efficacy of SCT‐1015 in mice bearing AMPKα1 silencing PLC5 xenograft tumors. Treatment with SCT‐1015 did not markedly inhibit the growth of AMPKα1 silencing PLC5 tumors (Fig. 7E) compared with PLC5 tumors with an empty vector. Our results demonstrated that AMPK is a key player in mediating the drug effects of SCT‐1015 *in vivo*.

### 3.7 The potential clinically relevant of SCT‐1015

To strengthen that the biological effect of SCT‐1015 in HCC cells is potentially clinically relevant, the growth inhibition of PLC5 cells under the treatment of SCT‐1015 and the other six compounds was compared. The six compounds were basically from three types of compounds. The first compound type exhibited the same target with SCT‐1015, such as two AMPK activators (A‐769662 and 991). The HIF1α inhibitor (PX‐478) and mTOR inhibitor (rapamycin) were selected as the second type, which involves the pathway related to AMPK. The third class is the first‐line approval drugs in HCC treatment, including sorafenib and lenvatinib. SCT‐1015 outperformed A‐769662, 991, PX‐478, and lenvatinib, and the IC_50_ of sorafenib (4.1 µm) was slightly lower than SCT‐1015 (7.4 µm) (Fig. 3A and Fig. S10A). Targeting multiple proteins in the same signaling pathway with different drugs exhibits a stronger on‐target inhibition or activation effect in the same pathway nodes, which may contribute to the longer duration of drugs or lower possibility for drug resistance [36]. We therefore validate whether SCT‐1015 exerts the potential on combined treatment with rapamycin, which represented the vertical inhibition in combinational strategies. Combined treatment of SCT‐1015 and rapamycin resulted in the markedly decreased viability in PLC5 cells and the significant retardation of tumor growth in PLC5‐bearing mice than each of the treatments alone (Fig. S10B and S11). These results supported the potential clinical application of SCT‐1015 in single‐treatment and combination therapy.

## 4 Discussion

The liver is the main organ for the control of glucose metabolism and energy homeostasis. When aberrant metabolic regulation occurs in the liver, normal hepatocytes show a high tendency to transform and result in several liver diseases, including HCC [37]. In HCC, cancer cells shift the metabolic status from OXPHOS to aerobic glycolysis to fulfill the ATP production and the demand of rapid cell growth. Such dependency of metabolic reprogramming significantly distinguishes cancer cells in HCC from their normal counterparts. Thus, targeting a metabolic enzyme or its upstream regulators constitute a feasible and attractive therapeutic approach in HCC treatment. In this study the newly developed nilotinib derivative, SCT‐1015, suppressed the flux of aerobic glycolysis through inhibiting hexokinase 2, adjusting the metabolic direction to enhance the OXPHOS status by prohibiting pyruvate dehydrogenase kinase 1, and subsequently retarding HCC growth *in vitro* and *in vivo* (Fig. S12). The inhibition of metabolic enzymes induced by SCT‐1015 resulted from the HIF1α protein degradation through AMPK activation. Our study demonstrated the potential application of AMPK activators in HCC treatment *via* reprogrammed metabolism.

Several metabolic enzymes have been found to participate in metabolic alteration during the process of hepatocarcinogenesis. For example, hexokinases catalyze the glucose metabolism to convert glucose to glucose 6‐phosphate, which is the first rate‐limiting step of aerobic glycolysis. In HCC, hexokinase 2 is the predominant hexokinase isoform [38]. Silencing hexokinase 2 reduced the ~ 40% and ~ 35% secretion rate of lactate and pyruvate, respectively, in Huh7 cells [27]. These results supported the key role of hexokinase 2 in controlling the glucose flux in HCC. However, controlling the upstream glycolytic flux may not be sufficient to explain the opposite downstream effects as increased lactate production and reduced mitochondrial oxidation. Pyruvate dehydrogenase is a gate‐keeping enzyme to determine the critical switch between glycolysis and the TCA cycle by converting pyruvate to acetyl‐coenzyme A (Fig. S12). The activity of pyruvate dehydrogenase is inhibited due to the highly expressed pyruvate dehydrogenase kinase 1 in HCC [39]. Inhibition of pyruvate dehydrogenase kinase 1 using a small molecule, dicoumarol, increased the OXPHOS flux in HCC, which can enhance the sensitivity of oxaliplatin for HCC chemotherapy [40]. SCT‐1015 provided the dual controlling point in metabolic reprogramming. One is the inhibition of hexokinase 2 to reducing the flux of glucose metabolism and suppressed the aerobic glycolysis. The other is targeting pyruvate dehydrogenase kinase 1 to reactivate pyruvate dehydrogenase and to direct pyruvate to acetyl‐coenzyme A instead of lactate. The dual targeting strategies of SCT‐1015 dramatically reduced the energy metabolism in HCC and channeled the HCC cells into an unfavored metabolic status (Fig. S12).

Under SCT‐1015 treatment, the protein level of HIF1α was reduced. Our results suggested that such a reduction was due to the increased protein degradation (ERK/PHD‐dependent proline hydroxylation on HIF1α) and the decline in protein synthesis (reduced mTORC1 activity) (Fig. S7,S8 and S12). In addition to the hydroxylation as the post‐translational modification, we have also found that HIF1α may be phosphorylated by p‐AMPK through protein complex formation. Previous studies have shown that the phosphorylation of glucose metabolism‐related proteins by AMPK resulted in the acceleration of protein degradation [41]. Transcription factors exert transacting roles to regulate the downstream genes for metabolism and development [42]. The stability of many human transcription factors is regulated by phosphorylation [43, 44]. Once the transcription factors are phosphorylated, they would be added with ubiquitin and then degraded [43, 44]. The protein degradation of the transcription factor phosphorylation can also be mediated by Lon protease [45]. Therefore, the potential phosphorylation of HIF1α by AMPK may also strengthen and accelerate the protein degradation. In other studies, transcription factor phosphorylation would cause the change of the subcellular localization, such as the translocation between the nucleus and cytoplasm [46, 47]. With the phosphorylation on the nucleus export signal, transcription factors would be exported out of the nucleus [48] and then lose the transacting ability for the downstream target genes. Either as enhanced protein degradation or the subcellular translocation, the phosphorylation on HIF1α may act synchronously with the proline hydroxylation to attenuate the function of HIF1α for the Warburg effect.

AMPK has been demonstrated to play a tumor suppressor role in cancer development. LKB1 and mammalian target of rapamycin (mTOR) are the most well‐known upstream and downstream regulators of AMPK, respectively [49]. The germline mutation of LKB1 showed high correlation with Peutz–Jeghers syndrome, usually coupled with multiple gastrointestinal hamartoma polyps [50]. mTOR is known to facilitate cancer cell growth and can be inhibited by AMPK [51]. Previous studies indicated that downregulation of the activity of AMPK or the enzymes in the AMPK signaling pathway exhibited high correlation with poor clinical outcomes in several cancer types. Two cohort studies indicated that the reduced AMPK signal was significantly associated with a higher histological grade and axillary node metastasis in primary breast cancer [52]. The reduced overall survival and recurrence‐free survival were observed in p‐AMPK‐negative nonsmall‐cell lung cancer patients [53]. In gastric cancer patients, the higher activity of cytoplasmic mTOR was associated with poorer overall survival and relapse‐free survival [54]. In this study, we developed an AMPK direct activator, SCT‐1015. Through the insertion to the pocket of α‐ and β‐subunits, SCT‐1015 released the kinase domain of α subunit to activate AMPK activity. With numerous clinical evidence, AMPK activators have been shown as valuable drugs for wide application in various cancer types. As a result, our SCT‐1015 exhibited strong therapeutic potential against many cancer types dependent on AMPK signaling.

Designing novel small molecule structures from a clinically available drug can provide lower potential for toxicity. We applied nilotinib (with both bcr‐abl inhibition and AMPK activation) as the lead compound for the design of a new AMPK activator. In comparison with the same concentrations of nilotinib treatment, the AMPK‐activating levels of nilotinib in our study (Fig. 1B) were consistent with that in the previous study [21]. The AMPK‐activating capacity of nilotinib was validated through a comparison of the nilotinib and the solvent control (DMSO). In the previous study, the concentrations of nilotinib were at 5 and 10 µm. In our study, four concentrations of nilotinib (5, 10, 15, and 20 µm) were used for demonstration. The same treatment concentrations (5 and 10 µm) were selected for evaluating the consistency of the activating capacity of nilotinib in the two studies. In these two concentrations, the activating capacities of nilotinib were not modest in Fig. 1B than that in the previous study. At the relatively high dose of nilotinib treatment (15 and 20 µm), the AMPK‐activating capacities were not improved along with the increasing concentration. When the concentration of nilotinib was high enough (20 µm), the activating capacity even showed a negative dose‐dependent correlation. We then hypothesized that the high concentration of nilotinib triggered some signals, which might cross‐interact or inhibit AMPK. To provide more specific structure characteristics on targeting AMPK, we removed the abl kinase inhibition from nilotinib to incorporate two novel advantages into the new AMPK activator, SCT‐1015. First, several abl kinase inhibitors caused cardiovascular toxicity [55, 56]. The enhancement of target (AMPK) specificity may reduce the excess risk of cardiovascular events. The application of our designed compound in cancer treatment can apply in the cancer cells growth that rely on AMPK but not abl. The second advantage is the application of our designed compound in combination with bcr‐abl inhibitors, such as nilotinib, in the treatment of chronic myelogenous leukemia (CML). Bcr‐abl inhibitors suppressed the bcr‐abl‐dependent mTOR signal to inhibit tumorigenesis, but the abl1 mutation and resistance to bcr‐abl inhibitors were observed in patients with CML [57]. The combination of SCT‐1015 (AMPK‐mTOR) and bcr‐abl inhibitors (bcr‐abl‐mTOR) would both suppress the mTOR signal through triggering different targets, which may reduce the toxicity and the resistance potential to abl inhibitor through the dose reduction of each drug.

Our design provides new information of the structure on developing a direct AMPK activator. Several domains or pockets of AMPK contain the possible interacting sites with small molecules. For example, the autoinhibitory domain of AMPKα is the interaction site of compound PT‐1, and such an interaction directly relieved the autoinhibition of AMPKα and increased AMPK activity [58]. SCT‐1015 directly interacted with AMPK through inserting into AMPKα‐ and β‐subunits to induce allosteric activation of AMPK. Although A‐769662 was also developed as an AMPK activator targeting the same binding pocket as SCT‐1015, some off‐target or side effects had been identified in the previous studies. For example, A‐769662 inhibited Na^+^‐K^+^‐ATPase independent of AMPK activation [59]. Such an off‐target effect altered the ion homeostasis and transmembrane currents, which may contribute to the complicated physiological effect of A‐769662. In another study, A‐769662 reduced the proteasome activity through an AMPK‐independent mechanism, which may increase the toxicity to mouse embryonic fibroblast cells [60, 61, 62]. The evidences were thought to contribute to the limited clinical utility of A‐769662 and also prompted the development of new AMPK activators for alternative options for cancer treatment. Our study provides new knowledge on AMPK activator development and showed the potential of a better safety profile through reducing off‐target effect in one small molecule.

## 5 Conclusion

SCT‐1015, a new structure entity developed based on nilotinib, allosterically activated AMPK through inserting into its α‐ and β‐subunits. SCT‐1015 was used to prove the concept of the alteration of the metabolic status *via* AMPK activation in HCC treatment. The AMPK‐dependent signaling triggered by SCT‐1015 downregulated HIF1α protein abundance to suppress the tumor progression in HCC cells and the xenograft mice model. In this study, our preclinical results strongly supported the future application of AMPK activators in HCC therapy.

## Conflict of interest

No conflicts of interest were declared.

## Author contributions

JCS designed the research; YSZ, HIT, MHL, JWH, CLL, FWY, TPF, ACM, and JCS performed the experiments and analyzed the data; YSZ, YJL, HIT, and JCS wrote the article.

## Supporting information


**Fig. S1**. Total synthesis of SCT‐1015.
**Fig. S2**. The spectrum of indicated compounds.
**Fig. S3**. The regulation of p‐AMPK complex *in vitro* by AMP and SCT‐1015.
**Fig. S4**. SCT‐1015 did not affect the activity of PP2A in PLC5 cells.
**Fig. S5**. SCT‐1015 induced cell death in a dose‐dependent manner in PLC5 cells.
**Fig. S6**. SCT‐1015 decreased the cellular ATP levels.
**Fig. S7**. SCT‐1015 suppressed the ERK axis in three HCC cells.
**Fig. S8**. The mTORC1 activity was inhibited by SCT‐1015.
**Fig. S9**. *In vivo* effects of SCT‐1015 on Huh7‐bearing mice.
**Fig. S10**. The cell viability of compounds in alone or combination treatment.
**Fig. S11**. The antitumor effect of the combination of SCT‐1015 and rapamycin *in vivo*.
**Fig. S12**. A drug mechanism scheme of SCT‐1015 on AMPK activation and metabolic reprogramming in HCC.

## Data Availability

The data that support the findings of this study are available from the corresponding author upon reasonable request.
