# Supplementary material for: A novel AMPK activator shows therapeutic potential in hepatocellular carcinoma by suppressing HIF1α‐mediated aerobic glycolysis
Source: Mol Oncol. 2022 Apr 12;16(11):2274–94. doi: 10.1002/1878-0261.13211 (PMC9168760; doi:10.1002/1878-0261.13211)

Supplementary Figure 1

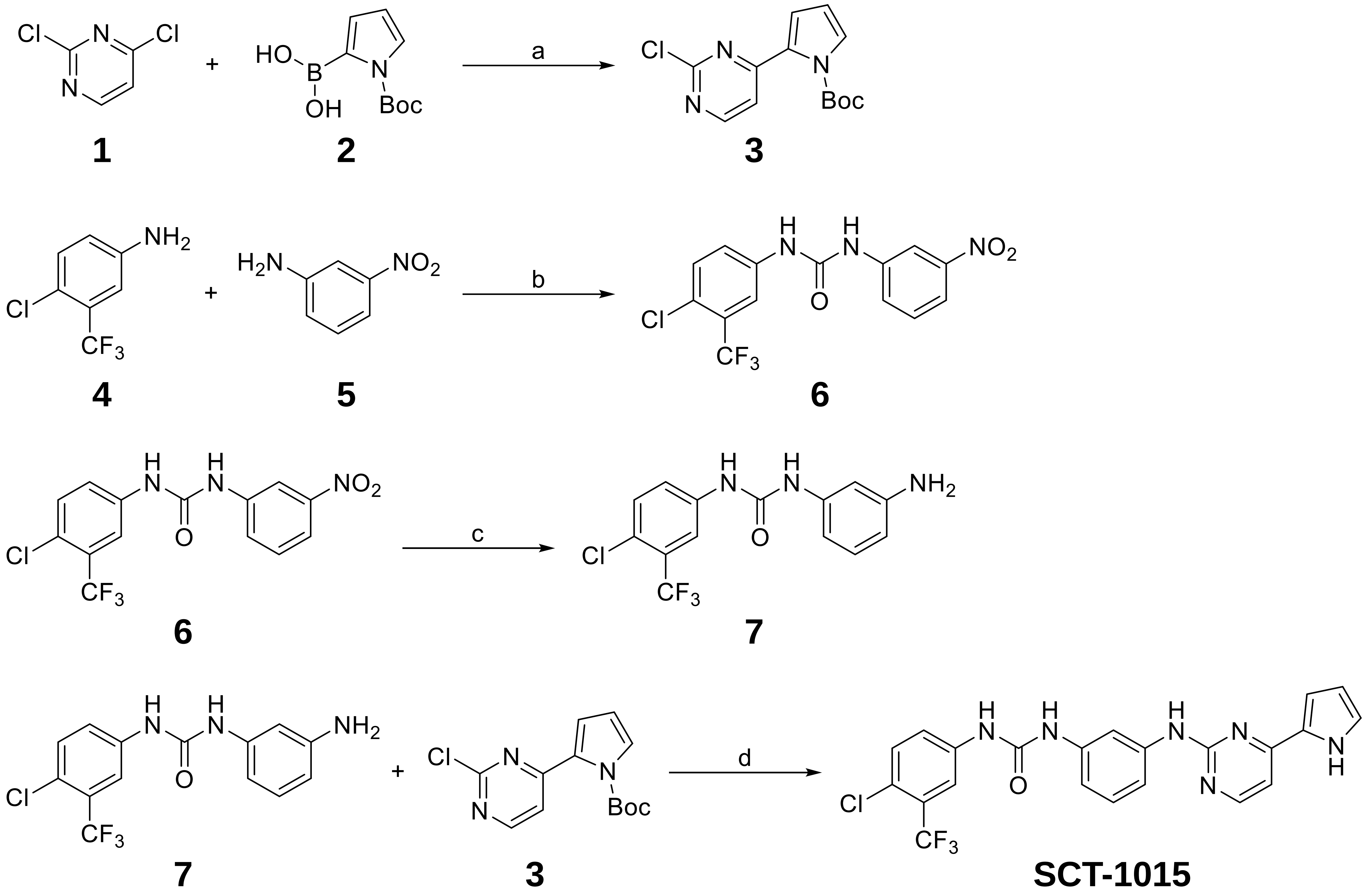

Supplementary Figure 2

A

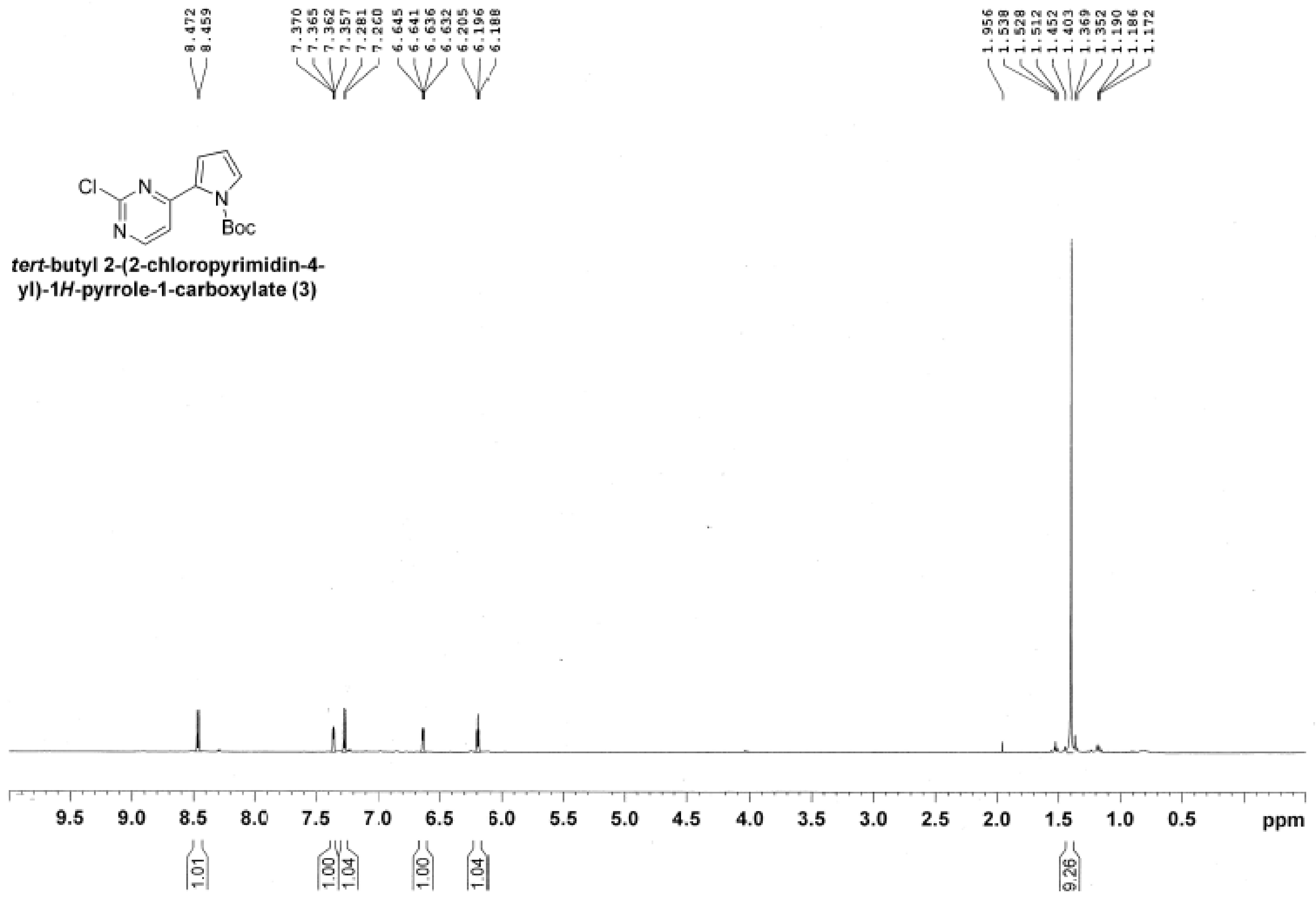

B

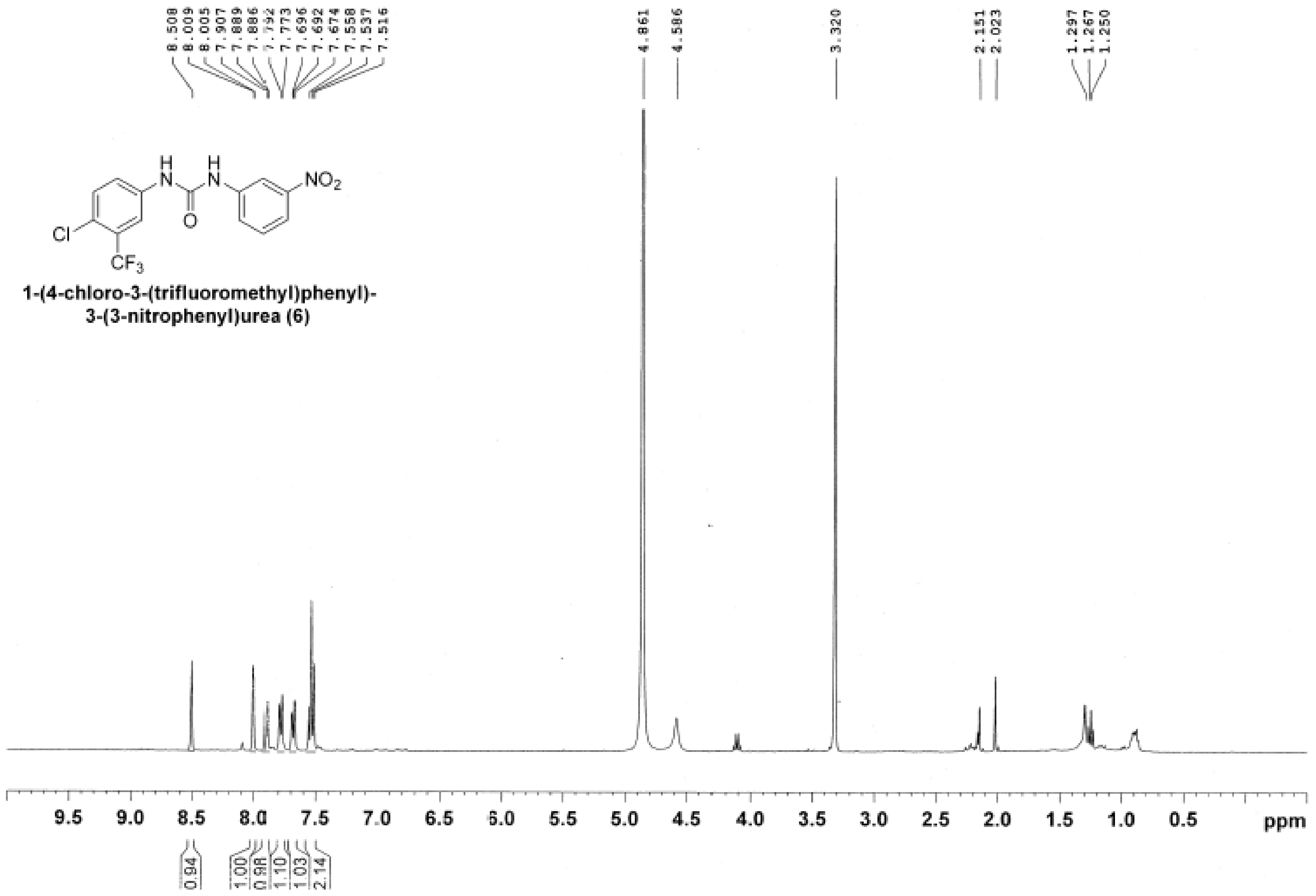

Supplementary Figure 2

C

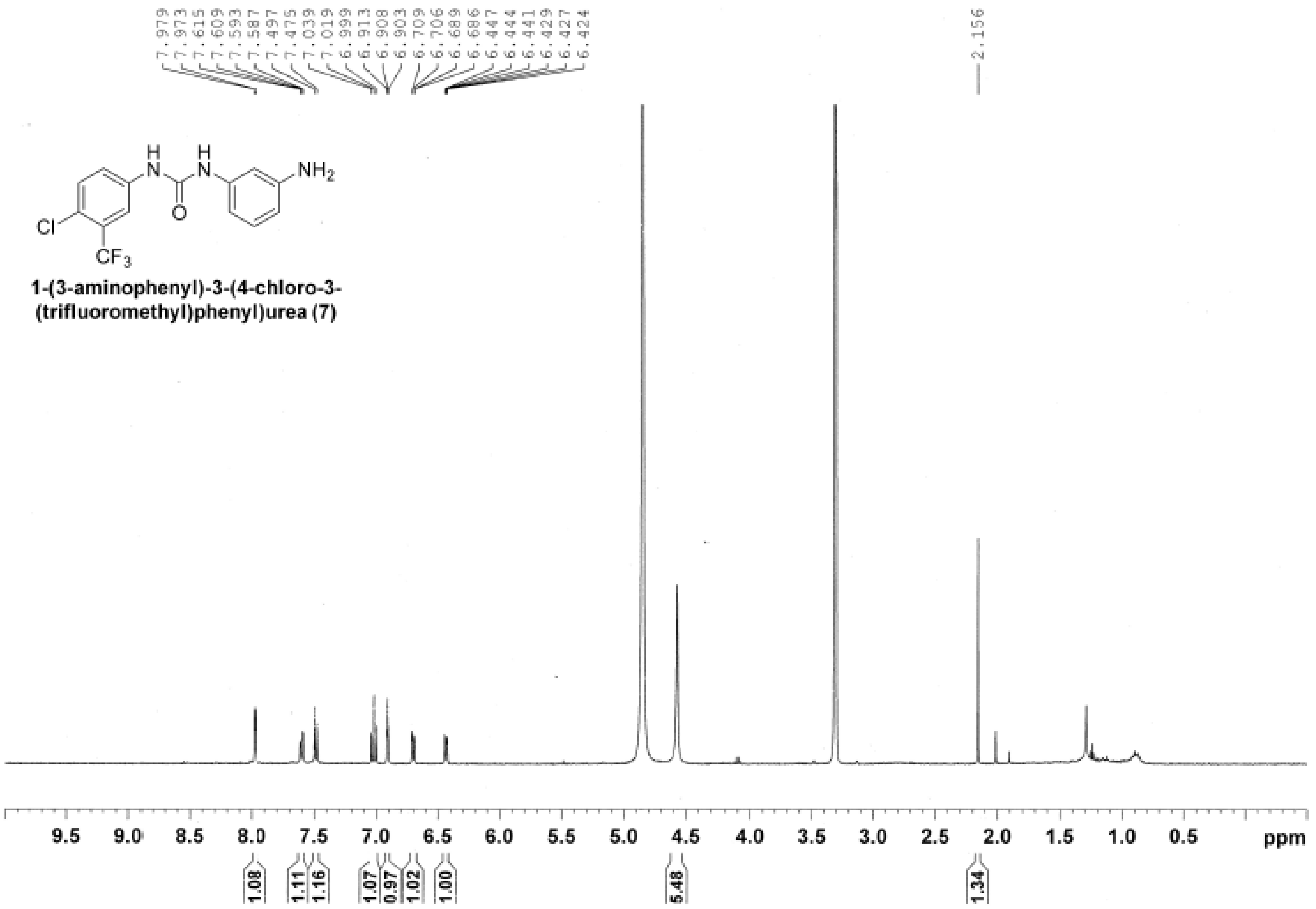

D

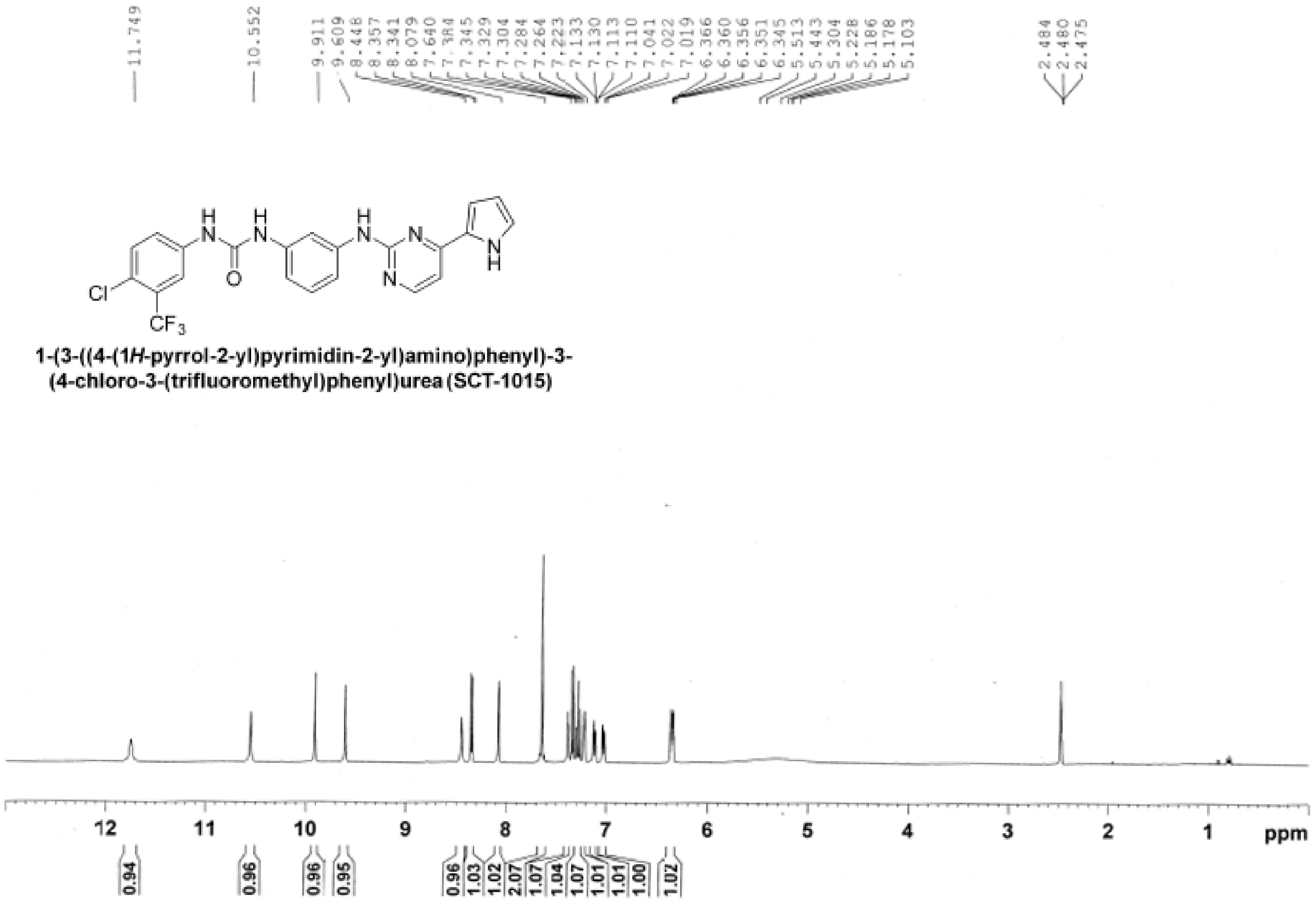

Supplementary Figure 2

E

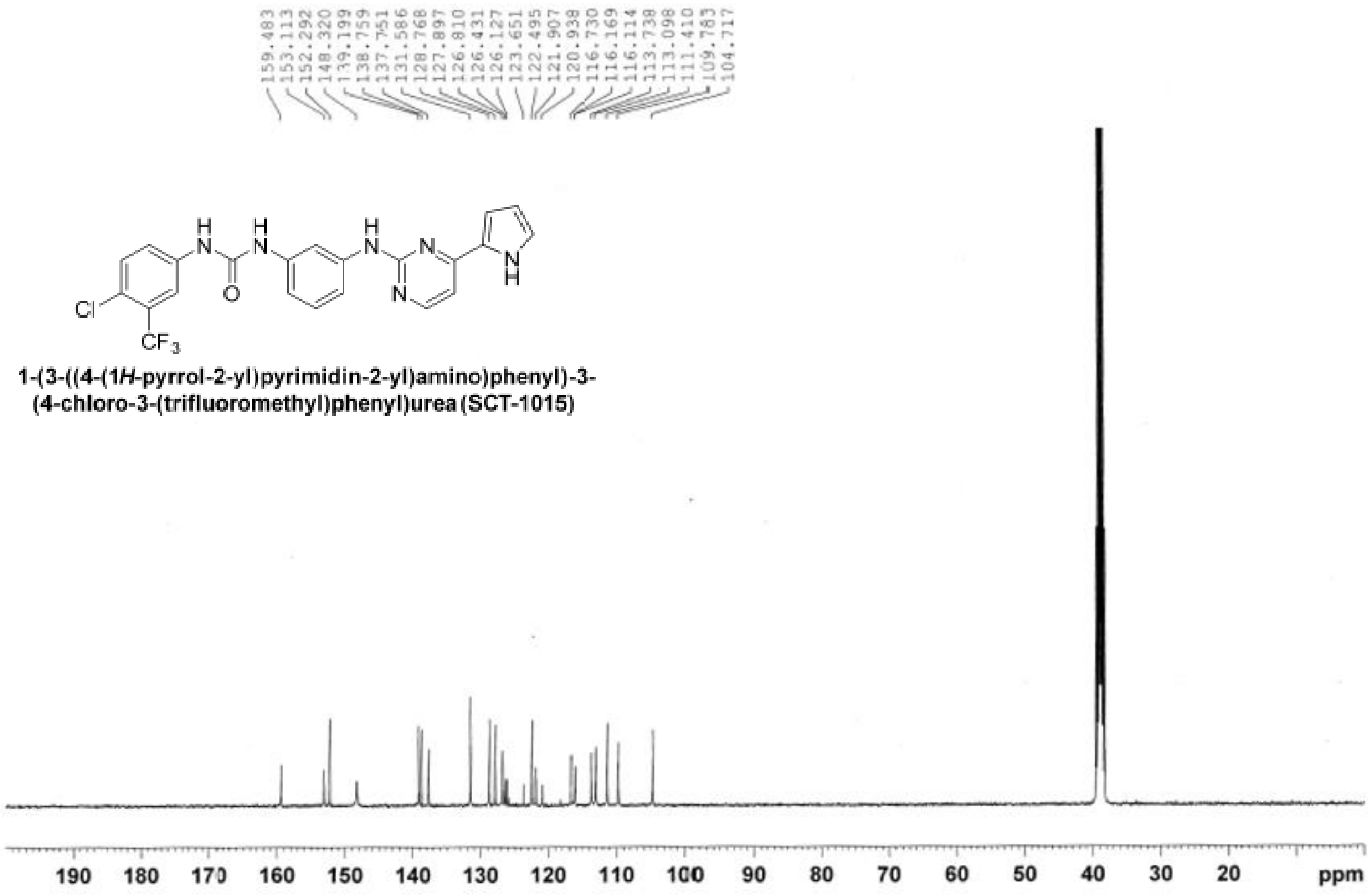

Supplementary Figure 3

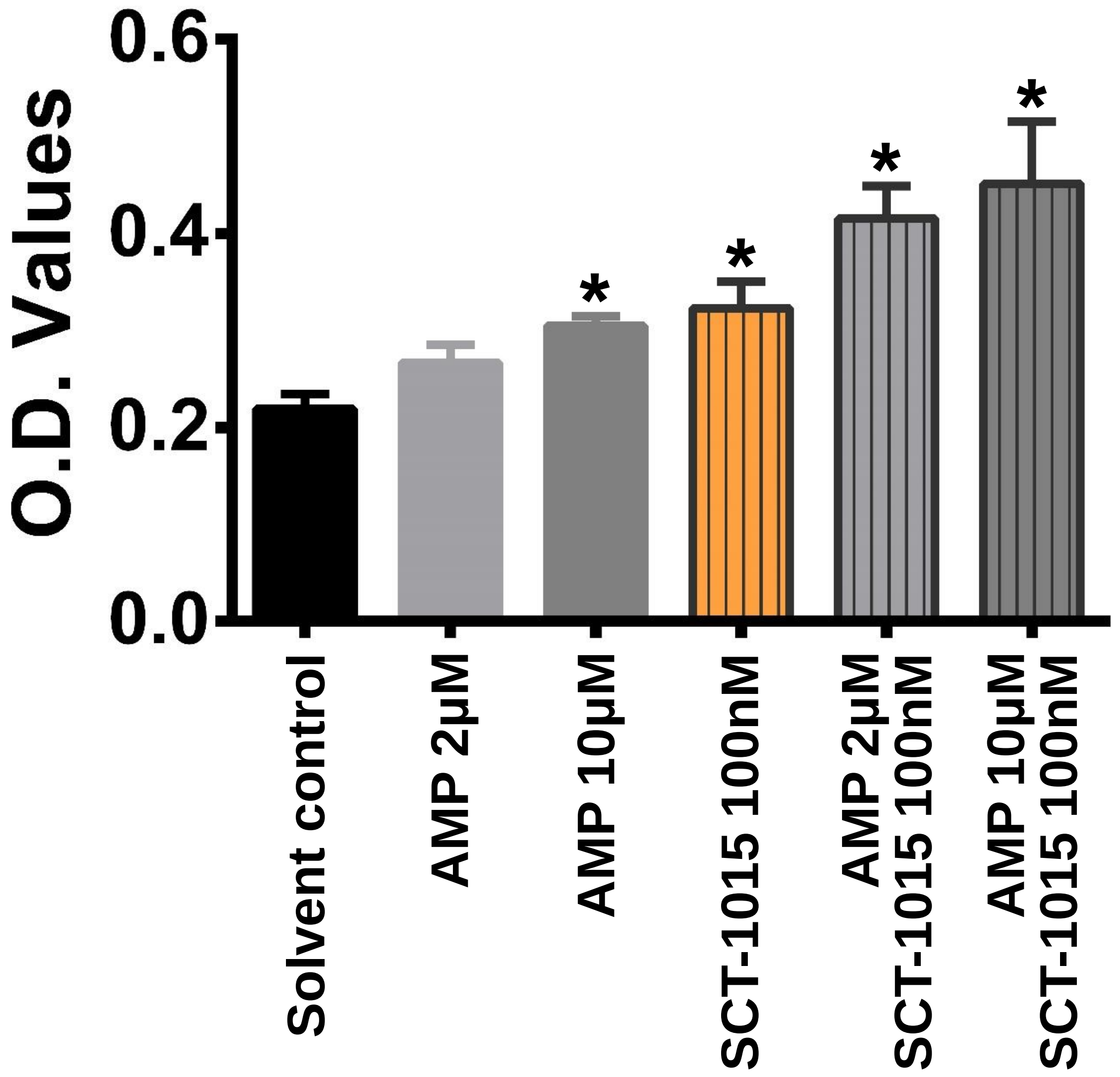

Supplementary Figure 4

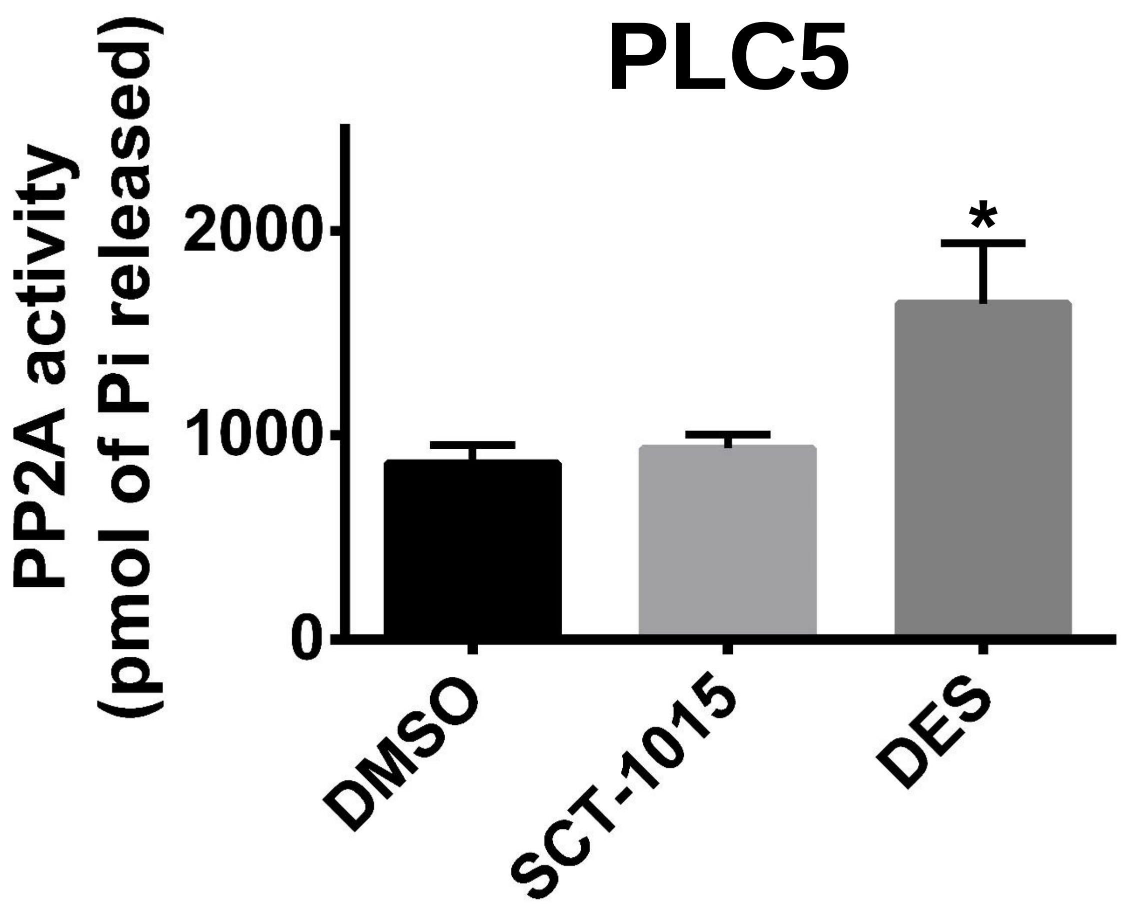

Supplementary Figure 5

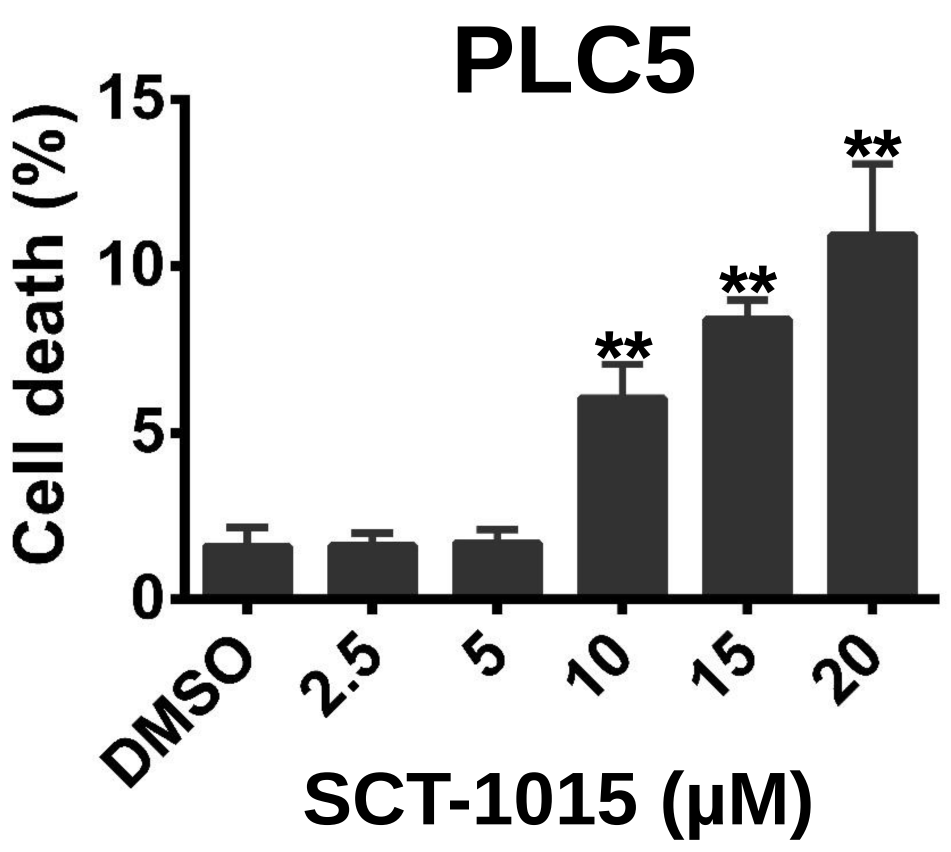

Supplementary Figure 6

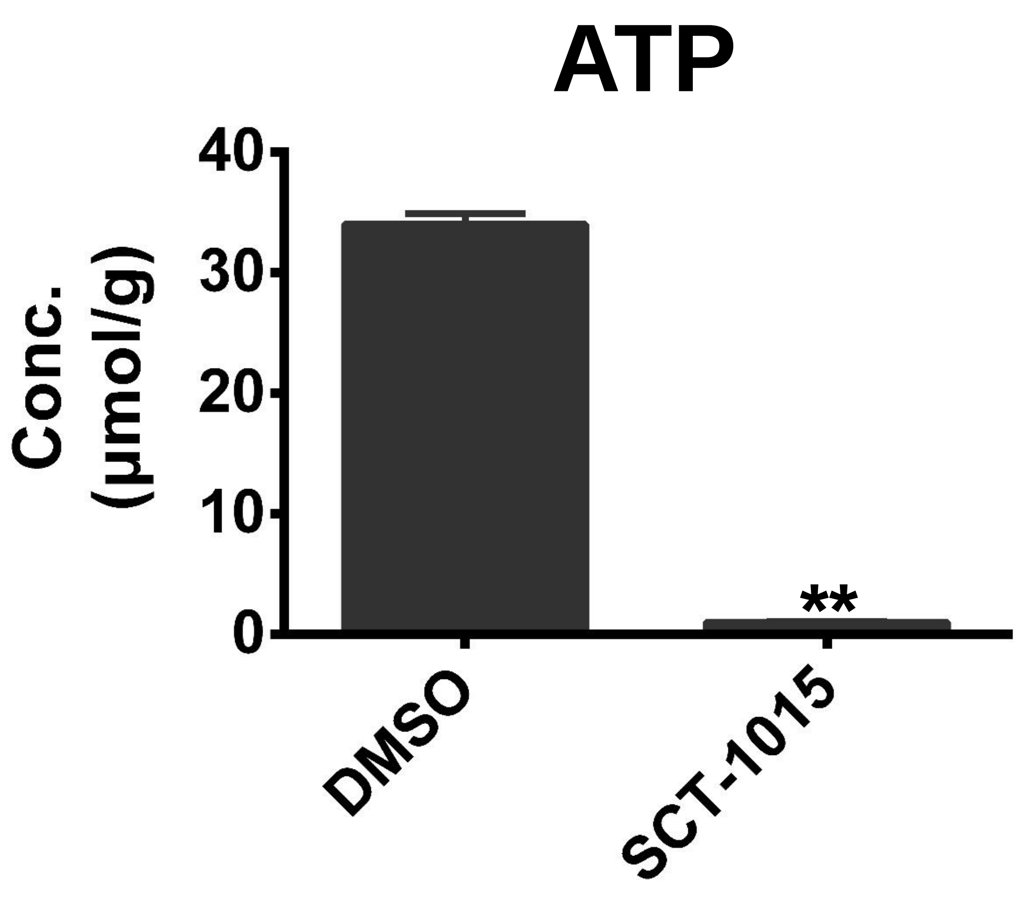

Supplementary Figure 7

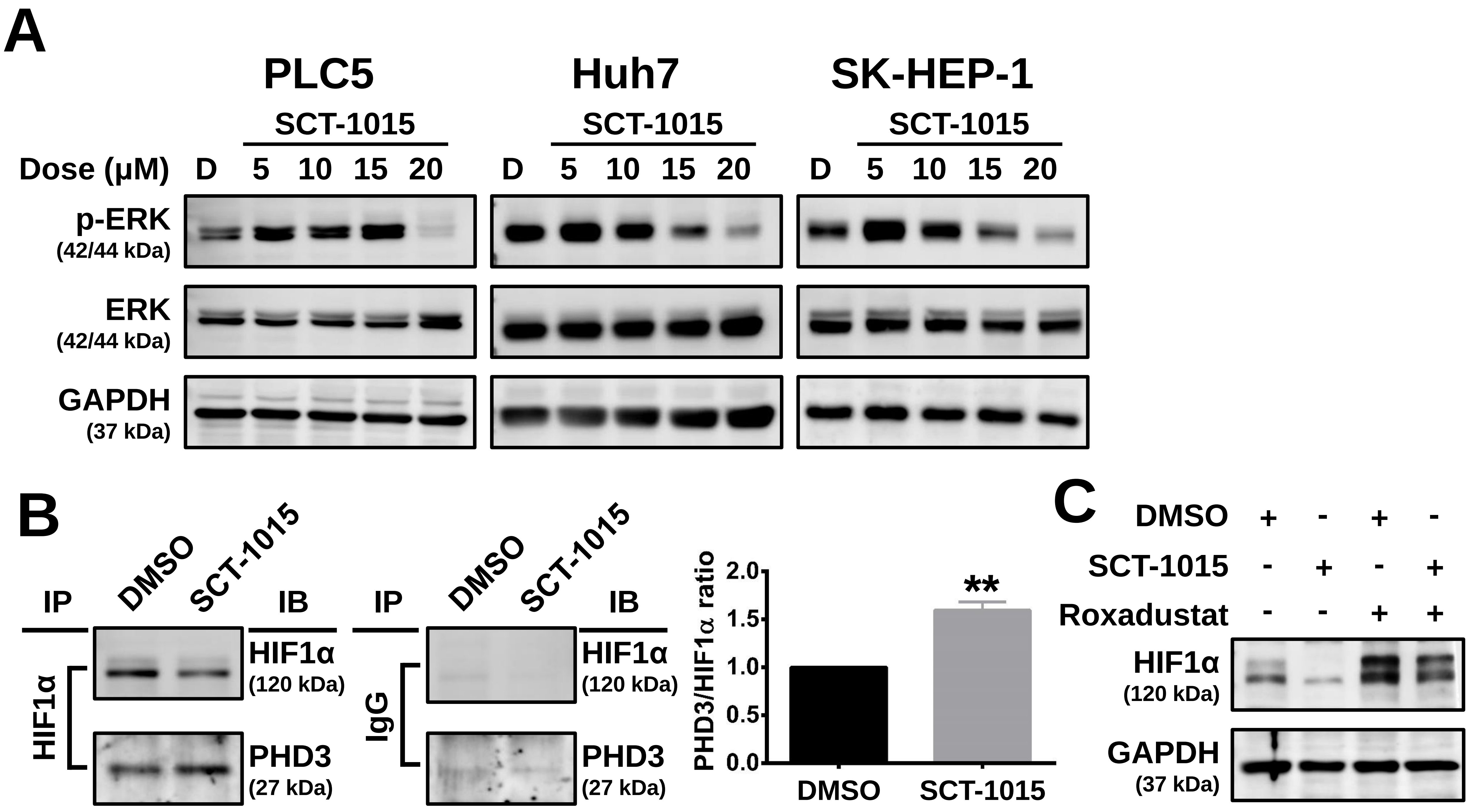

Supplementary Figure 8

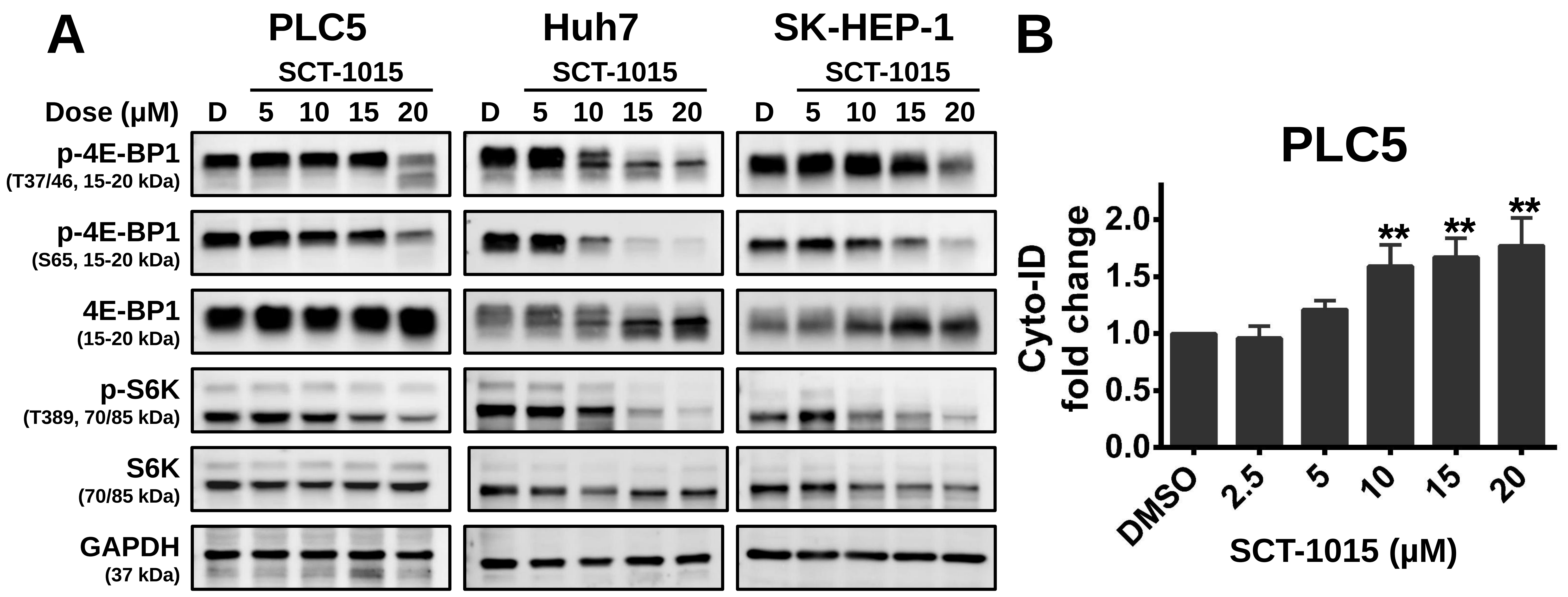

Supplementary Figure 9

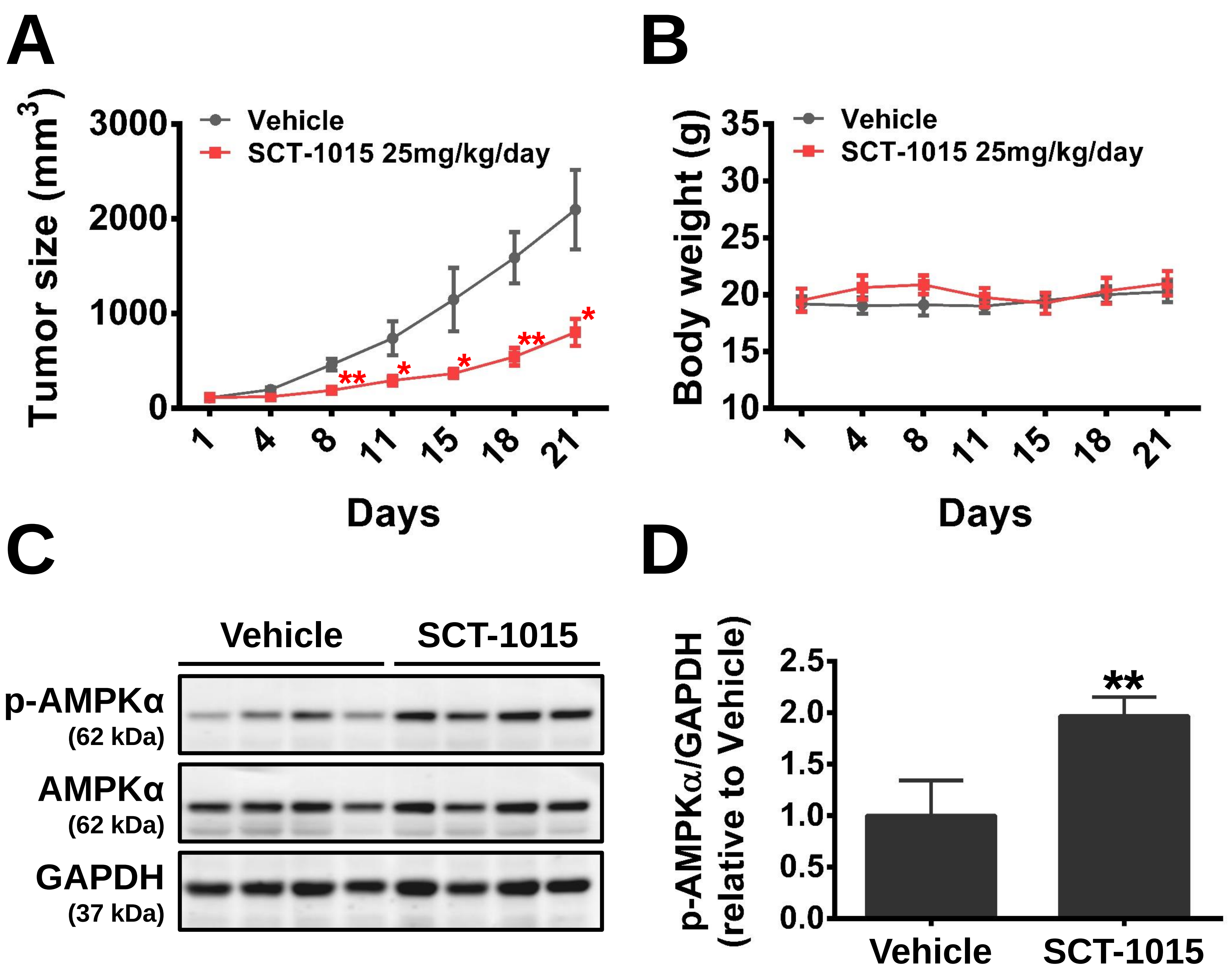

Supplementary Figure 10

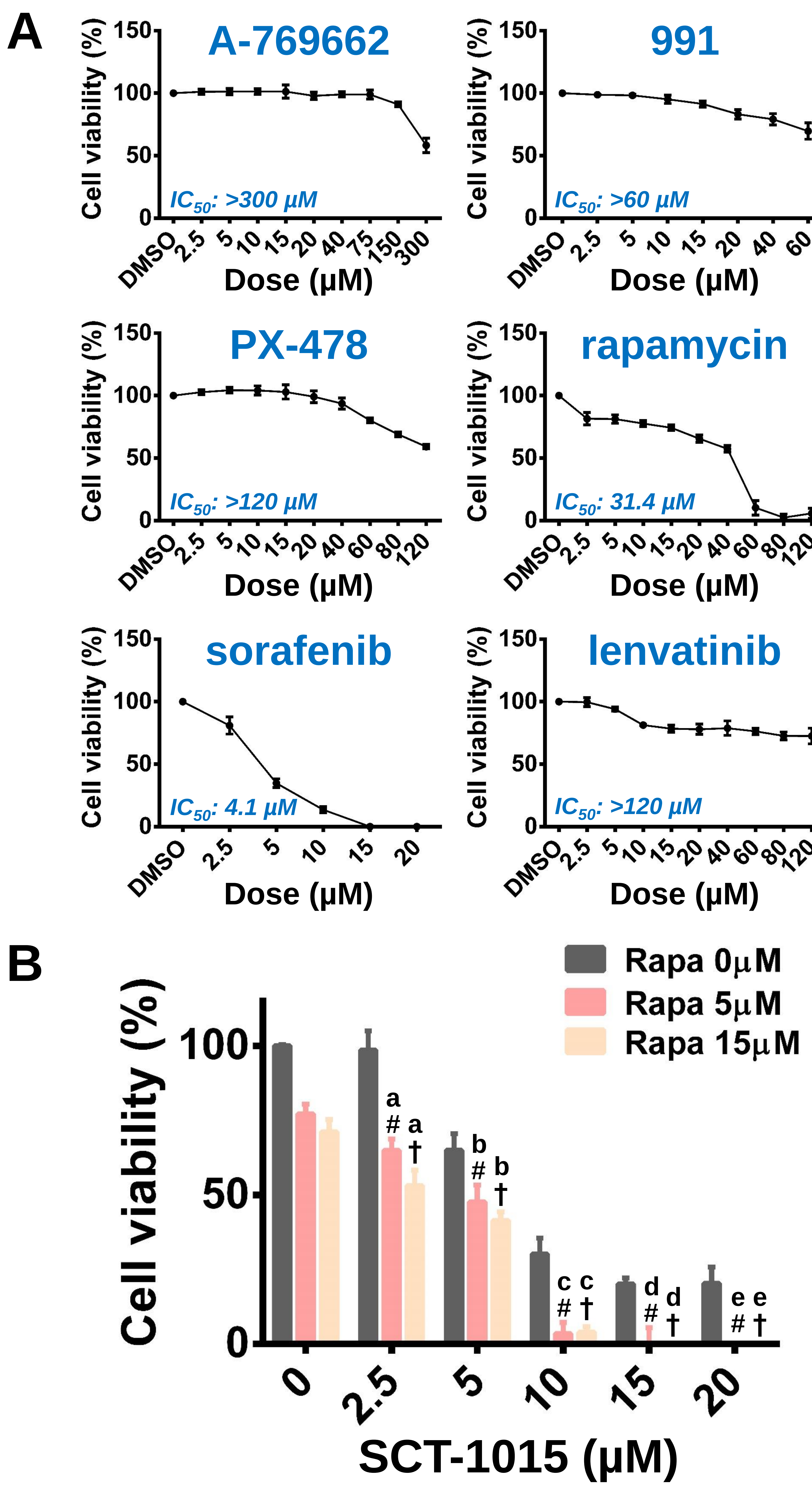

Supplementary Figure 11

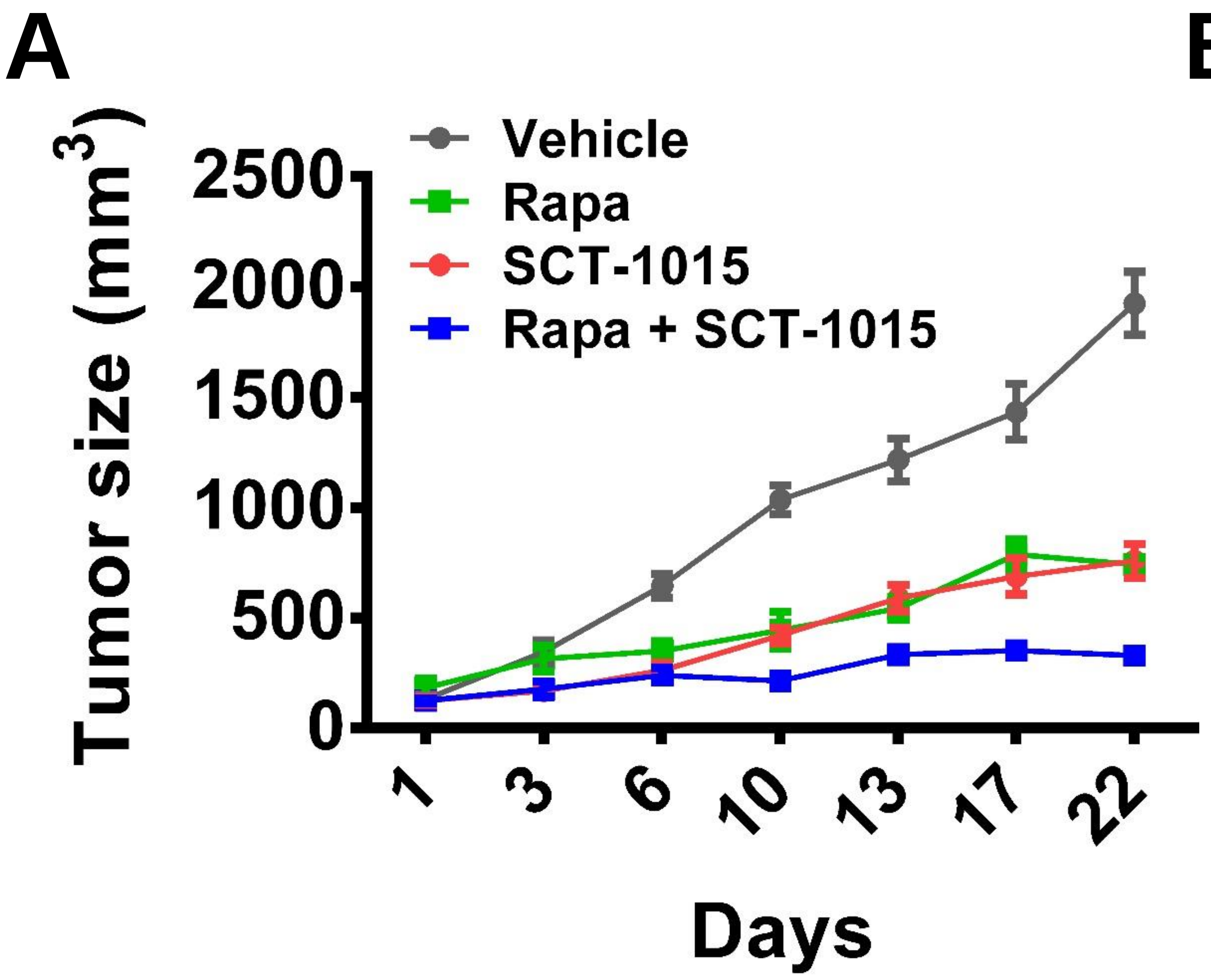

Day 22 Rapa vs. Vehicle (*p*<0.0001)
SCT-1015 vs. Vehicle (*p*<0.0001)
Rapa+SCT-1015 vs. Vehicle (*p*<0.0001)
Rapa vs. Rapa+SCT-1015 (*p*=0.0107)
SCT-1015 vs. Rapa+SCT-1015 (*p*=0.0077)

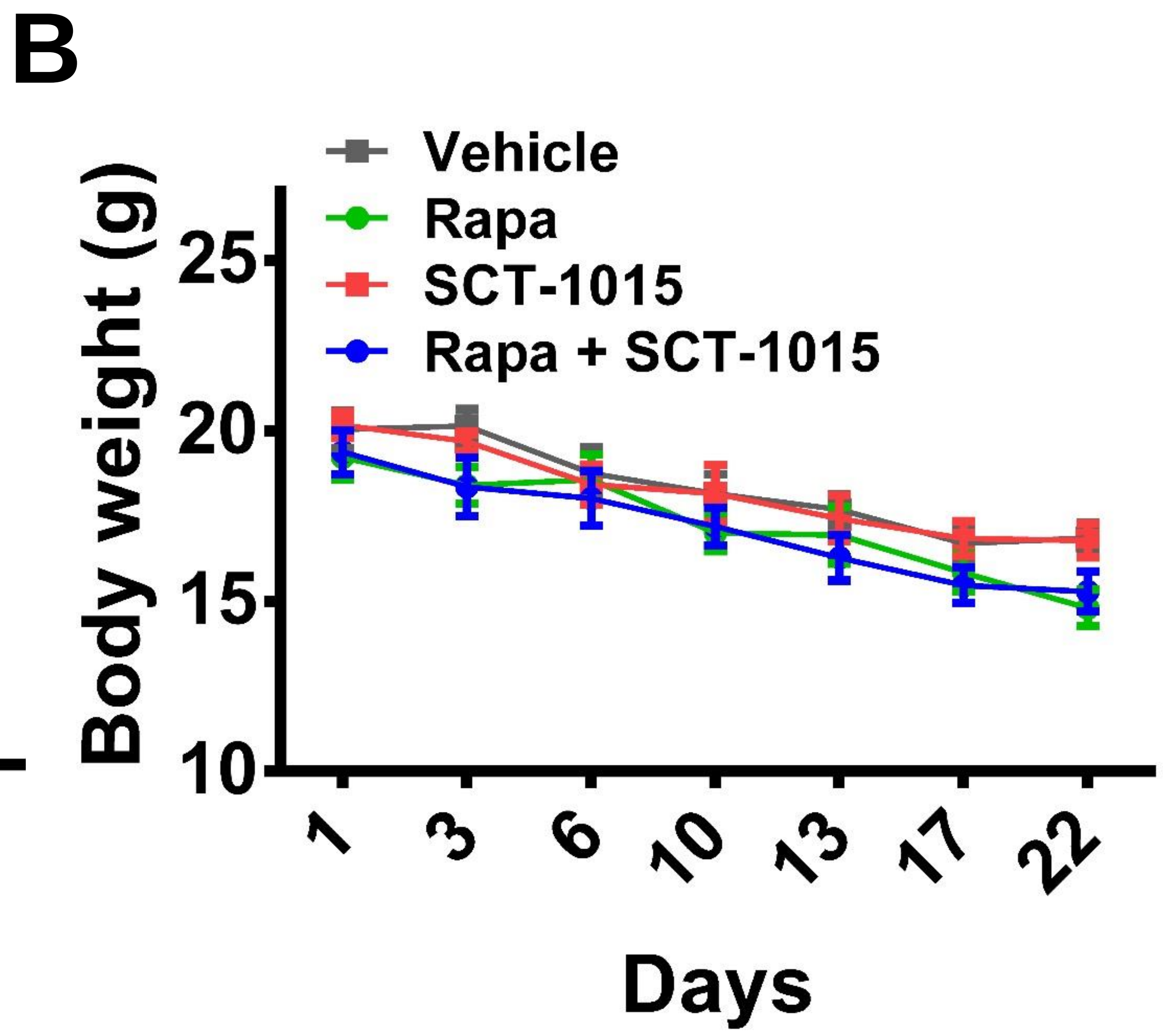

Day 22 Rapa vs. Vehicle (*p*=0.048)
SCT-1015 vs. Vehicle (N.S.)
Rapa+SCT-1015 vs. Vehicle (N.S.)
Rapa vs. Rapa+SCT-1015 (N.S.)
SCT-1015 vs. Rapa+SCT-1015 (N.S.)

Supplementary Figure 12

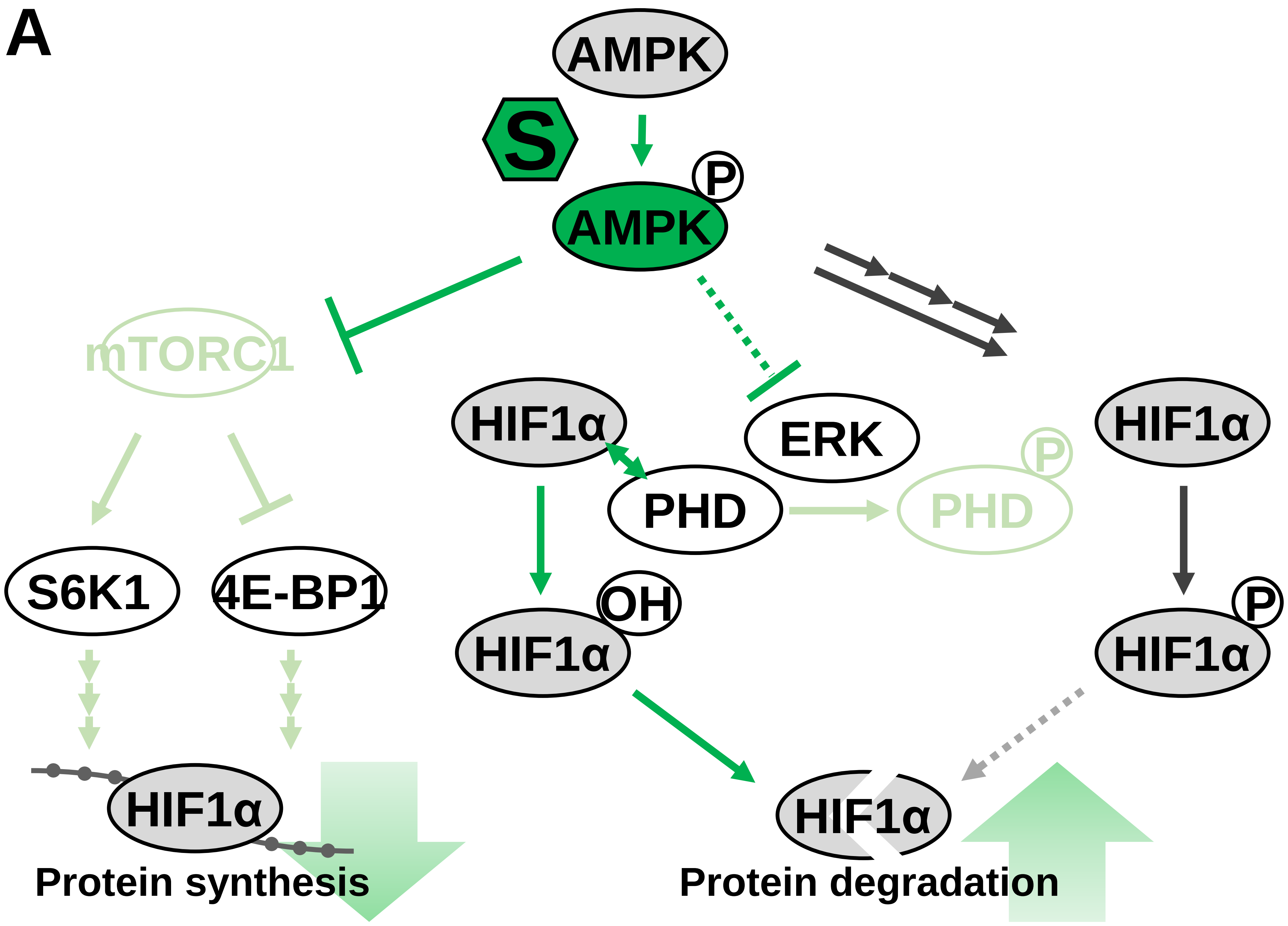

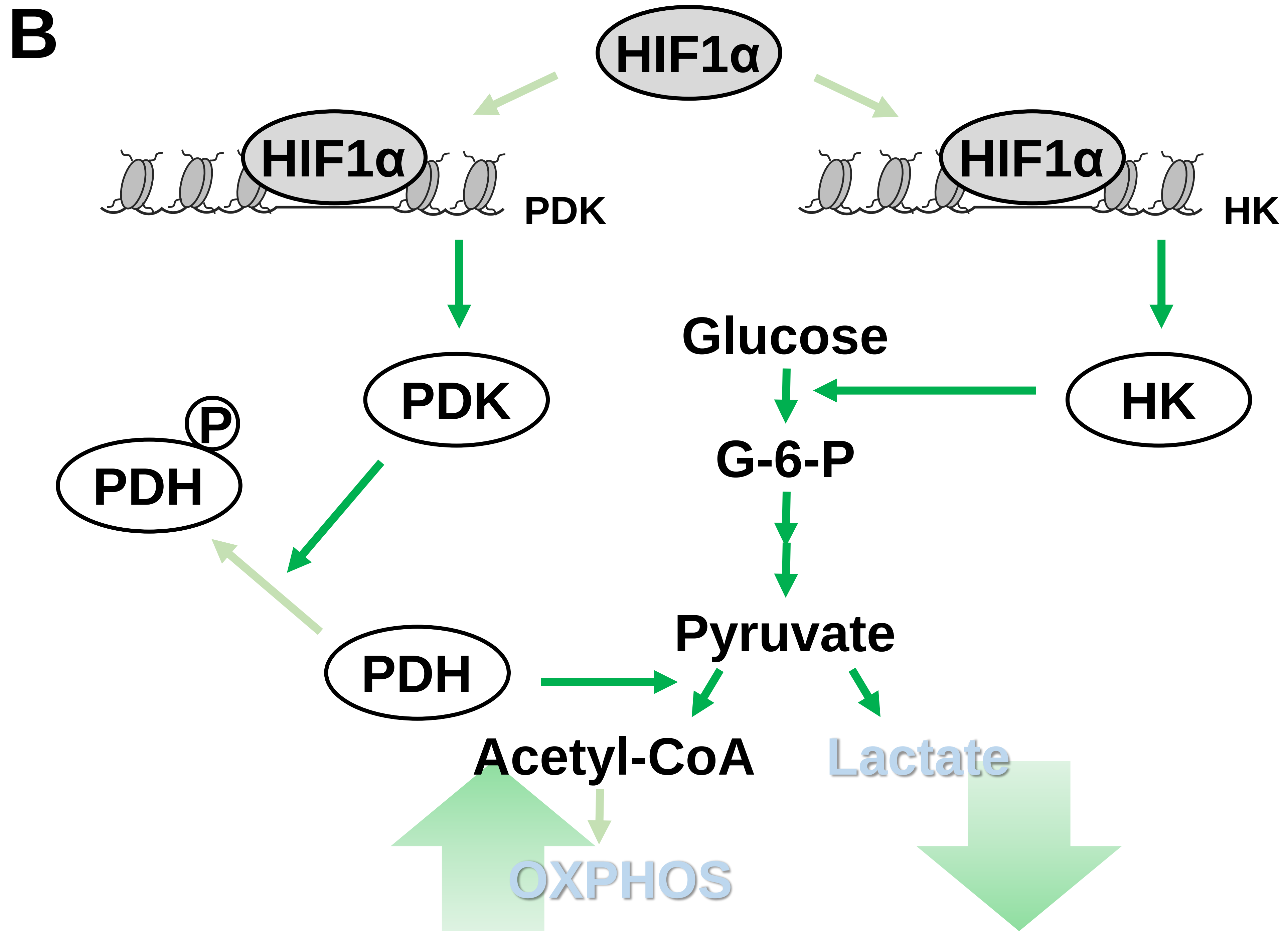

Supplement: Supplementary file 1 — Fig. S1. Total synthesis of SCT‐1015. Fig. S2. The spectrum of indicated compounds. Fig. S3. The regulation of p‐AMPK complex in vitro by AMP and SCT‐1015. Fig. S4. SCT‐1015 did not affect the activity of PP2A in PLC5 cells. Fig. S5. SCT‐1015 induced cell death in a dose‐dependent manner in PLC5 cells. Fig. S6. SCT‐1015 decreased the cellular ATP levels. Fig. S7. SCT‐1015 suppressed the ERK axis in three HCC cells. Fig. S8. The mTORC1 activity was inhibited by SCT‐1015. Fig. S9. In vivo effects of SCT‐1015 on Huh7‐bearing mice. Fig. S10. The cell viability of compounds in alone or combination treatment. Fig. S11. The antitumor effect of the combination of SCT‐1015 and rapamycin in vivo. Fig. S12. A drug mechanism scheme of SCT‐1015 on AMPK activation and metabolic reprogramming in HCC. [file MOL2-16-2274-s001.pdf]
